# Diagnostic and prognostic value of long noncoding RNAs as biomarkers in urothelial carcinoma

**DOI:** 10.1371/journal.pone.0176287

**Published:** 2017-04-21

**Authors:** Johanna Droop, Tibor Szarvas, Wolfgang A. Schulz, Christian Niedworok, Günter Niegisch, Kathrin Scheckenbach, Michèle J. Hoffmann

**Affiliations:** 1 Department of Urology, Medical Faculty, Heinrich-Heine-University Duesseldorf, Duesseldorf, Germany; 2 Department of Urology, Medical Faculty, University of Duisburg-Essen, Essen, Germany; 3 Department of Urology, Semmelweis University Budapest, Budapest, Hungary; 4 Department of Otolaryngology, Medical Faculty, Heinrich-Heine-University Duesseldorf, Duesseldorf, Germany; Centro Nacional de Investigaciones Oncologicas, SPAIN

## Abstract

Many long noncoding RNAs (lncRNAs) are deregulated in cancer and contribute to oncogenesis. In urothelial carcinoma (UC), several lncRNAs have been reported to be overexpressed and proposed as biomarkers. As most reports have not been confirmed independently in large tissue sets, we aimed to validate the diagnostic and prognostic value of lncRNA upregulation in independent cohorts of UC patients. Thus, expression of seven lncRNA candidates (GAS5, H19, linc-UBC1, MALAT1, ncRAN, TUG1, UCA1) was measured by RT-qPCR in cell lines and tissues and correlated to clinicopathological parameters including follow-up data (set 1: N n = 10; T n = 106). Additionally, publicly available TCGA data was investigated for differential expression in UC tissues (set 2: N n = 19; T n = 252,) and correlation to overall survival (OS). All proposed candidates tended to be upregulated in tumour tissues, with the exception of MALAT1, which was rather diminished in cancer tissues of both data sets. However, strong overexpression was generally limited to individual tumour tissues and statistically significant overexpression was only observed for UCA1, TUG1, ncRAN and linc-UBC1 in tissue set 2, but for no candidate in set 1. Altered expression of individual lncRNAs was associated with overall survival, but not consistently between both patient cohorts. Interestingly, lower expression of TUG1 in a subset of UC patients with muscle-invasive tumours was significantly correlated with worse OS in both cohorts. Further analysis revealed that tumours with low TUG1 expression are characterized by a basal-squamous-like subtype signature accounting for the association with poor outcome. In conclusion, our study demonstrates that overexpression of the candidate lncRNAs is found in many UC cases, but does not occur consistently and strongly enough to provide reliable diagnostic or prognostic value as an individual biomarker. Subtype-dependent expression patterns of lncRNAs like TUG1 could become useful to stratify patients by molecular subtype, thus aiding personalized treatments.

## Introduction

Urothelial carcinoma (UC) represents the most common histological subtype of urinary bladder cancer. It is a heterogeneous disease, ranging from low-malignant tumours restricted to the urothelial tissue layer (stage pTa) to highly malignant and often lethal muscle-invasive carcinomas (≥pT2). Notably, less malignant variants of UC tend to recur quite frequently and high-grade lower stage tumours may progress to invasive cancers. For these reasons, improved diagnostic and prognostic biomarkers are desirable, especially to monitor for recurrences, to identify patients with a poor prognosis and to select appropriate treatments for individual patients [1].

Long noncoding RNAs (lncRNAs) are a diverse set of transcripts, which are defined as encompassing more than 200 nucleotides, but do not contain any substantial open reading frame. Many lncRNAs are expressed in a tissue-specific fashion and have been reported to undergo expression changes during tumour development or progression [2]. These properties render them valid candidates as diagnostic or prognostic cancer biomarkers. In addition, lncRNAs appear to be functionally involved in oncogenesis exerting tumour-promoting or tumour-suppressive functions [2,3].

Several lncRNAs have been reported to be frequently or consistently overexpressed in UC and have been proposed as individual diagnostic or prognostic biomarkers; some were moreover demonstrated to influence proliferation, survival, migration and other cancer-relevant properties of UC cell lines [4]. These lncRNAs include UCA1 [5], MALAT1 [6], H19 [7], GAS5 [8], TUG1 [9], ncRAN (SNHG16) [10] and linc-UBC1 (BLACAT) [11]. However, only few lncRNAs have been validated independently for their suitability as biomarkers in different populations or for their functional involvement in UC.

For instance, some studies have concurrently reported overexpression of HOXC11-AS, also known as HOTAIR, in many UCs, which appears to be associated with higher grade and worse prognosis. In cell culture experiments, HOTAIR likewise often confers a more aggressive phenotype [12,13]. In contrast, the lncRNA MEG3 encoded in an imprinted cluster at 14q32 is strongly down-regulated in most UCs according to two independent reports [14,15]. The requirement for replication studies in this field is further illustrated by the case of the lncRNA CDKN2B-AS, also known as ANRIL, which was proposed to be upregulated in UC in one study, but reported as essentially unchanged in another one [16,17]. In addition to differences in the study populations, this discrepancy may relate to the investigation of different ANRIL isoforms between the two studies, since a third investigation found frequent upregulation of specific splice variants only [18].

In the present study, we have therefore investigated the expression of the seven candidate lncRNAs mentioned above, for which insufficient validation data has been published so far. We used RT-qPCR on total RNA from 12 UC cell lines compared to a benign cell line and a large tissue set with complete clinical and histopathological data. Where appropriate, we initially used different primer pairs to check whether transcript variants of the respective lncRNA differ in their expression across UC cell lines. Expression data from tissues was compared with results obtained from the TANRIC database [19], which provides convenient access to the RNA-seq data and clinical information from the comprehensive investigation of UC genomics by the TCGA consortium. These data were analysed to determine for each lncRNA whether it was more strongly expressed in cancer tissues than in benign tissues, whether its expression correlated with important histopathological parameters, and especially whether any association with clinical outcome could be discerned.

## Material and methods

### Patients and tissues

The set of tissue samples (set 1) used for quantitative real time RT-PCR analysis (RT-qPCR) consisted of 106 tumour tissues and 10 benign tissues. The majority of the patients was diagnosed with muscle-invasive and high-grade bladder cancer (13 pTa, 13 pT1, 15 pT2, 44 pT3, 19 pT4; 39 low-grade, 67 high-grade). This tissue set was collected according to the principles expressed in the Declaration of Helsinki and with written patient informed consent as approved by the ethics committee of the medical faculty of the University Duisburg-Essen, Study Number 07–3537. A further amendment was approved by the committee allowing the extensive characterization of such tissues including the analysis of tissue-RNA by RT-PCR. Median follow up time for the complete cohort was 22.7 months (range 0.2–198).

Sample preparation and expression analyses were performed and described in accordance with the MIQE guidelines (minimum information for publication of quantitative real time PCR experiments) as detailed in the supplementary methods section (S1 File) and supplementary S1–S3 Tables.

Expression data provided for lncRNAs by the TANRIC database is based on the TCGA bladder urothelial carcinoma (BLCA) dataset (set 2) consisting of 252 tumour tissue samples and 19 benign tissue samples, which were with only few exceptions obtained from muscle-invasive tumours (one pT1, 72 pT2, 118 pT3, 38 pT4, 23 without information about staging). Median follow-up time for this cohort was 15.9 months (0.49–163).

Analyses of molecular subtype gene expression signatures were performed by means of the cBioPortal tool based on the provisional TCGA bladder urothelial carcinoma set (“extended” data set: 413 tumors in total; 408 tumor samples with RNA Sequencing mRNA expression (3 pTa, 121 pT2, 195 pT3 and 59 pT4) and Genesis 1.0 software.

### Cell lines

UC cell lines VM-CUB1, SW-1710, HT-1376, 5637, and BFTC-905 were obtained from the DSMZ (Braunschweig, Germany), the cell lines T-24, RT-4, RT-112, 639-V, J82, UM-UC-3 and UM-UC-6 were kindly made available by Dr. J. Fogh (New York, NY), Dr. M. A. Knowles (Leeds, UK) and Dr. B. Grossman (Houston, USA) [20]. Cell lines were regularly verified by DNA fingerprint analysis and checked for mycoplasm contamination. The TERT-immortalized normal human urothelial cell line TERT-NHUC was obtained from Dr. M. A. Knowles [21] and cultured in keratinocyte serum-free medium (Gibco, life technologies; Carlsbad, CA, USA) supplemented with 0.25 ng/ml epidermal growth factor, 12.5 μg/ml bovine pituitary extract and 1:100 ITS (Gibco), 0.35 μg/ml (-)N-epinephrine and 0.33 mg/ml hydrocortisone (Sigma Aldrich; St. Louis, MO, USA).

### RNA extraction and reverse transcription

RNA was extracted using Qiazol reagent (Qiagen; Hilden, Germany) and chloroform and precipitated by isopropanol. It was further purified by means of the RNeasy Mini Kit (Qiagen) including on-column DNAse digestion. Quality and integrity of the RNA were assessed on 1.5% agarose gels. Tissue RNA was reverse transcribed using the High Capacity cDNA Reverse Transcription Kit (Applied Biosystems; Foster City, CA, USA). RNA from cell lines was reverse transcribed by means of the Quantitect reverse transcription Kit (Qiagen) according to the manufacturer.

### Quantitative real time PCR

RT-qPCR analyses in set 1 for the seven lncRNAs and the proliferation marker gene *MKI67* as an additional control were performed on a Roche Lightycler 96 (Roche; Risch, Switzerland). Reactions were carried out in 20 μl volume using the QuantiTect SYBR Green RT-PCR Kit (Qiagen) with 10 pmol of each primer. Information on primer sequences, annealing temperatures and standard curves can be obtained from S1 Table. Reactions comprised an initial activation step for 15 minutes at 95°C and 40 cycles each with denaturation at 94°C for 20 s, annealing at an individual temperature for 20 s and DNA synthesis at 72°C for 30 s, followed by a final melting curve analysis. Concentration values are calculated based on standard curves carried out for each gene and each run using the Roche Lightcycler 96 software (Version 1.1). PCR efficiency and melting peak integrity was checked for each run. Each sample was assayed in duplicates. Specific run information on slope, efficiency, coefficient of determination (R^2) and mean of melting temperature across all samples (Tm) is given in S2 and S3 Tables. *TBP* and *SDHA* were measured as reference genes [22] and a normalization factor was calculated for each sample using their geometric mean [23]. Expression values of the assessed lncRNAs and *MKI67* are given relative to this normalization factor. See supplementary methods (S1 File) for further information about RT-qPCR conditions according to MIQE guidelines.

### Use of the TANRIC and cBioPortal database analysis tools

The TANRIC database provides easy access to publicly available RNA-Seq data for various tumour entities, especially for lncRNA expression. In the TANRIC database lncRNA expression was analyzed for the TCGA bladder urothelial carcinoma (BLCA) dataset and queried in the “My lnRNA”-tool by either lncRNA annotation or, for *linc-UBC1* [chr1:205404014–205425214] and [chr17:74553846–74561430] for *ncRAN*, in hg19 genomic coordinates. LncRNA expression values were obtained as log2 RPMK (reads per kilo base per million mapped reads) from the TANRIC database as indicated in the “My lncRNA”-tool and median expression was calculated for benign and tumour samples and visualized in boxplots using R. Additional boxplots and Kaplan-Meier curves for each lncRNA were obtained from the database tool.

The cBioPortal database tools (Oncoprint, download of mRNA Expression z-Scores (RNA Seq V2 RSEM)) were employed to explore correlations between expression of lncRNA and marker genes defining molecular subtypes of UC.

### Statistical methods

Mann–Whitney U test was performed for paired group comparisons. Overall survival and disease-specific survival analysis was done using univariate Cox regression analysis. In addition Kaplan–Meier log-rank test was applied for overall survival. For multivariate analysis, the Cox proportional hazards regression model was applied and variables with effect on survival in univariate analysis (p ≤ 0.05) were included in the Cox proportional hazards regression models. A p-value of at most 0.05 was considered to be statistically significant. All statistical analyses were performed with the SPSS software package (version 21; SPSS).

## Results

### Expression changes of candidate lncRNAs in UC and their correlation with clinicopathological parameters

**Fig 1:**
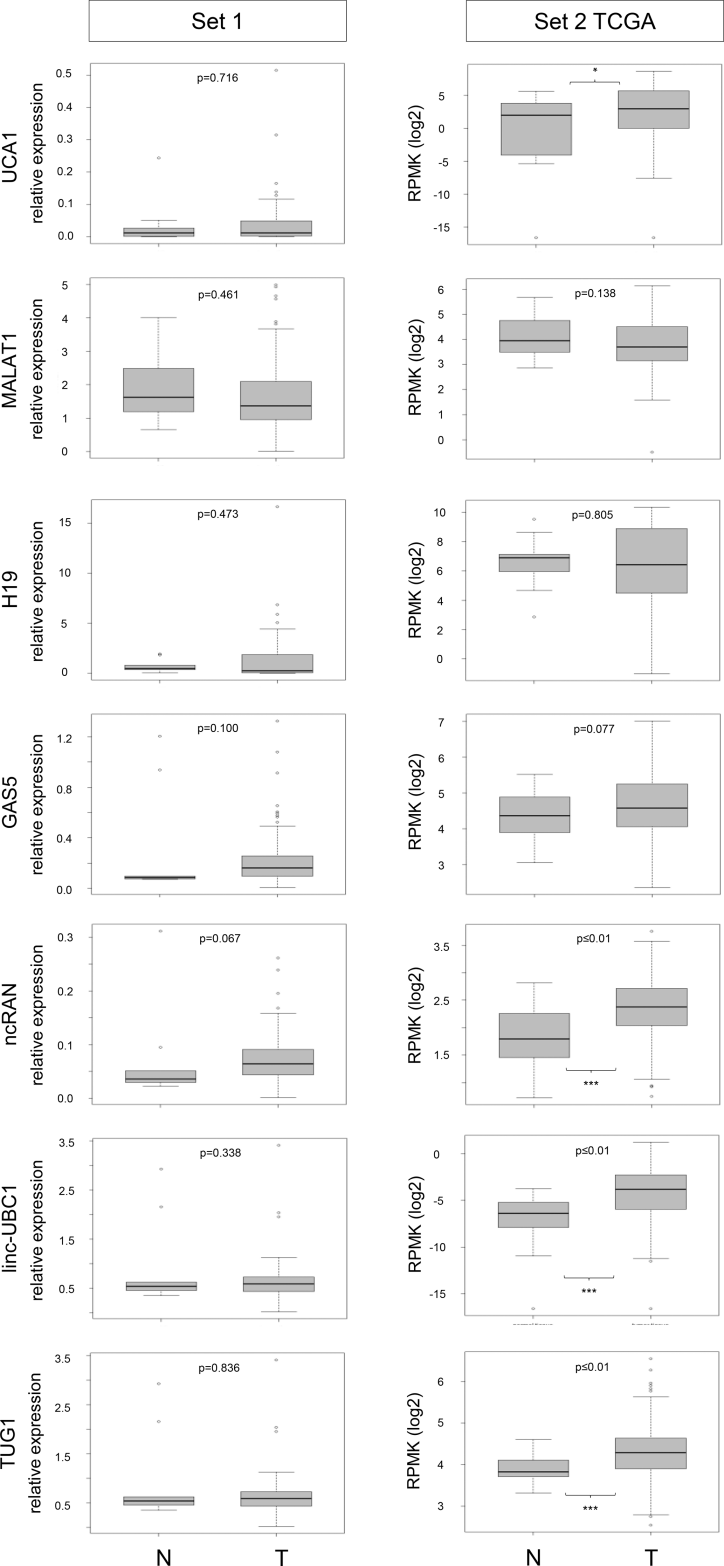
lncRNA expression data for tumour and benign tissues from tissue set 1 and 2. Boxplot representations of lncRNA expression in set 1 (RT-qPCR, relative expression to geometric mean of reference genes *SDHA* and *TBP*) and set 2 (RNA-Seq in the TCGA bladder cancer cohort, expression as log2 RPMK, data obtained from the TANRIC database). P-values for difference between control (N) and tumour (T) samples were calculated by Mann-Whitney U-test.

Expression of the seven lncRNAs *UCA1*, *MALAT1*, *H19*, *GAS5*, *TUG1*, *ncRAN* (*SNHG16*) and *linc-UBC1* (*BLACAT*) was measured by RT-qPCR in UC tissue set 1 consisting of 106 tumor tissues and 10 benign tissues obtained from radical cystectomies. The results are summarized as boxplots depicting relative expression in tumour and benign tissues for each lncRNA (Fig 1, left). For the same lncRNAs, expression data from the TCGA RNA-sequencing study of 252 UC tissues (set 2) was evaluated via the TANRIC database. Boxplots for comparison between tumour and benign tissues are likewise presented in Fig 1 (right). For sample set 1 we additionally measured the expression of the proliferation marker gene *MKI67* (encoding Ki67). As expected, *MKI67* was weakly expressed in most benign tissues and highly significantly overexpressed in the tumour samples (S1 Fig).

The RT-qPCR results on set 1 indicated no difference in the median expression of *UCA*1 between normal and tumour tissues (Fig 1 left, median 1.19 vs. 1.2). Likewise, only 2/12 investigated UC cell lines displayed *UCA1* overexpression compared to control cells (S2 Fig). In the TCGA data (set 2, Fig 1 right), a slight (1.5-fold in median expression), but statistically significant (p = 0.026) increase in *UCA1* expression in tumour tissues was observed (Fig 1). Although a previous report on suitability of *UCA1* as a urine biomarker stated that high expression of *UCA1* is associated with high grade and high stage of UC [3], the TCGA data rather indicates that increased *UCA1* expression originated mainly from higher *UCA1* expression levels in non-muscle invasive UC (NMIBC) compared to muscle-invasive disease (MIBC; S3 Fig right). Similarly, *UCA1* expression tended to be higher in non-invasive and in low-grade tumours rather than muscle-invasive tumours of set 1 (S3 Fig left), but these differences were not statistically significant (Table 1).

**Table 1:** Clinical and histopathological parameters of tissue set 1.

**Variables**		UCA1 *100		UBC1 *100		TUG1 *10		ncRAN *100		MALAT1 *10		H19 *10		GAS5 *10	
															
	n	median (range)	P	median (range)	P	median (range)	P	median (range)	P	median (range)	P	median (range)	P	median (range)	P
**Age**															
**≤ 65**	54	1.04 (0.00–51.55)	0.184	7.58 (0.00–111.83)	0.937	5.88 (0.19–19.52)	0.752	5.80 (0.14–26.16)	0.072	16.53 (0.06–49.94)	0.300	3.74 (0.03–68.49)	0.582	1.53 (0.22–13.27)	0.383
**> 65**	52	1.43 (0.02–31.48)		6.07 (0.00–3361.76)		5.88 (2.67–34.14)		7.70 (0.55–23.92)		12.93 (4.14–45.59)		2.10 (0.06–166.48)		1.79 (0.05–10.79)	
**Gender**															
**Male**	76	1.50 (0.00–31.48)	0.337	7.83 (0.00–194.30)	0.257	6.06 (2.58–20.41)	0.326	6.53 (0.55–26.16)	0.736	13.99 (4.54–49.94)	0.088	3.59 (0.03–68.49)	0.068	1.66 (0.22–10.79)	0.643
**Female**	30	0.41 (0.01–51.55)		5.74 (0.00–3361.76)		5.00 (0.19–34.14)		5.96 (0.14–14.77)		11.39 (0.06–36.73)		0.81 (0.06–166.48)		1.54 (0.05–13.27)	
**Stage**															
**Ta**	13	1.57 (0.00–9.58)	0.801	18.48 (0.00–194.30)	0.479	6.51 (2.67–8.90)	0.243	6.39 (0.78–14.43)	0.186	18.95 (7.03–49.25)	0.579	7.97 (0.16–68.49)	0.139	1.75 (0.55–9.13)	0.545
**T1**	13	1.15 (0.04–11.61)	0.902	6.88 (0.84–101.54)	0.229	7.11 (3.73–20.41)	0.341	7.07 (3,12–16.79)	0.059	16.77 (4.14–38.91)	0.742	0.92 (0.13–35.62)	0.805	1.86 (0.65–10.79)	0.113
**T2**	17	1.25 (0.04–16.52)	0.949	15.31 (1.22–180.38)	**0.004**	5.81 (2.82–11.25)	0.688	4.38 (1.32–15.30)	0.104	16.81 (6.97–33.65)	0.143	1.85 (0.03–29.44)	0.077	1.65 (0.32–6.05)	0.652
**T3**	44	1.57 (0.02–51.55)	0.077	4.31 (0.00–111.83)	0.193	5.58 (2.58–9.20)	0.237	6.35 (0.55–26.16)	0.611	11.84 (3.37–45.70)	0.323	4.54 (0.06–166.48)	0.214	1.44 (0.46–4.93)	0.309
**T4**	19	0.28 (0.01–12.84)		8.45 (0.05–3361.71)		5.85 (0.19–34.14)		5.33 (0.14–23.92)		13.68 (0.06–49.94)		1.31 (0.10–58.70)		2.15 (0.05–13.27)	
**Non-inv.**	26	1.36 (0.00-11-61)	0.552	9.27 (0.00–194.30)	0.331	6.58 (2.67–20.41)	0.419	6.92 (0.78–16.79)	0.318	17.55 (4.14–49.25)	**0.050**	4.33 (0.13–68.49)	1.000	1.85 (0.55–10.79)	0.052
**Invasive**	80	1.19 (0.01–51.55)		6.33 (0.00–3361.76)		5.78 (0.19–34.14)		6.16 (0.14–26.16)		12.65 (0.06–49.94)		2.57 (0.03–166.48)		1.51 (0.05–13.27)	
**Grade**															
**G1**	7	2.37 (0.01–9.58)	0.707	9.49 (1.62–55.40)	0.530	6.84 (2.81–8.90)	0.680	6.93 (0.78–15.88)	0.872	16.84 (7.03–46.59)	0.680	8.41 (0.16–68.49)	0.484	2.78 (0.68–3.89)	0.872
**G2**	32	1.48 (0.00–11.61)	0.600	14.93 (0.00–194.30)	**0.002**	6.35 (2.67–11.25)	0.098	7.42 (1.32–23.92)	0.125	14.49 (4.14–49.25)	0.887	2.21 (0.03–58.70)	0.708	1.74 (0.32–9.13)	0.540
**G3**	67	1.12 (0.01–51.55)		3.72 (0.00–3361.76)		5.52 (0.19–34.14)		5.93 (0.14–26.16)		13.58 (0.06–49.94)		2.58 (0.06–166.48)		1.55 (0.05–13.27)	
**Low-grade (G 1–2)**	39	1.57 (0.00–11.61)	0.489	14.55 (0.00–194.30)	**0.001**	6.51 (2.67–11.25)	0.065	7.07 (0.78–23.92)	0.150	15.04 (4.14–49.25)	0.736	3.13 (0.03–68.49)	0.930	1.75 (0.32–9.13)	0.441
**High-grade (G 3)**	67	1.12 (0.01–51.55)		3.72 (0.00–3361.76)		5.52 (0.19–34.14)		5.93 (0.14–26.16)		13.58 (0.06–49.94)		2.58 (0.06–166.48)		1.55 (0.05–13.27)	
**Lyph node**															
**N0/Nx/M0/Mx**	78	1.47 (0.00–51.55)	0.576	7.66 (0.00–2261.8)	0.717	5.78 (2.67–34.14)	0.562	6.64 (0.78–19.58)	0.552	14.28 (4.14–49.94)	0.971	2.51 (0.03–166.44)	0.903	1.57 (0.05–10.79)	0.333
**N + / M+**	28	1.03 (0.01–16.52)		5.09 (0.00–111.83)		6.14 (0.19–10.04)		5.70 (0.14–26.16)		13.38 (0.06–45.70)		3.22 (0.06–58.70)		1.75 (0.33–13.27)	
**Smoking**															
**no**	29	1.60 (0.01–51.55)	0.511	6.87 (0.24–194.30)	0.257	6.56 (0.19–20.41)	0.214	8.48 (0.14–23.92)	0.238	12.69 (0.06–45.70)	0.589	2.35 (0.06–58.70)	0.292	2.03 (0.46–13.27)	0.130
**yes**	58	0.90 (0.00–31.48)		5.63 (0.00–111.83)		5.82 (2.58–9.66)		6.95 (0.78–26.16)		13.67 (4.14–49.25)		4.00 (0.08–166.48)		1.78 (0.05–9.13)	
**unknown**	19														
**Control**	10	1.19 (0.02–24.41)	0.716	2.87 (0.13–6217.9)	0.338	5.36 (3.47–29.25)	0.836	3.63 (2.29–31.20)	0.067	16.26 (6.54–40.11)	0.461	4.94 (0.42–19.40)	0.473	0.85 (0.71–12.07)	0.100
**Tumor**	106	1.20 (0.00–51.55)		6.67 (0.00–3361.76)		5.88 (0.19–34.14)		6.46 (0.14–26.16)		13.67 (0.06–49.94)		2.60 (0.03–166.48)		1.60 (0.05–13.27)	

Information on patient characteristics is given in relation to expression levels of the analysed lncRNAs (median expression relative to the geometric mean of the reference genes *SDHA* and *TBP*, UCA1*100 indicates that the expression values were multiplied with 100 for better interpretation of the data). P-values were calculated for differences between age groups, gender, non-invasive vs invasive, T stage, low grade vs. high grade, metastatic status, smoking habits and control vs. tumour. Bold printed p-values are significant (≤0.05).

**Fig 2:**
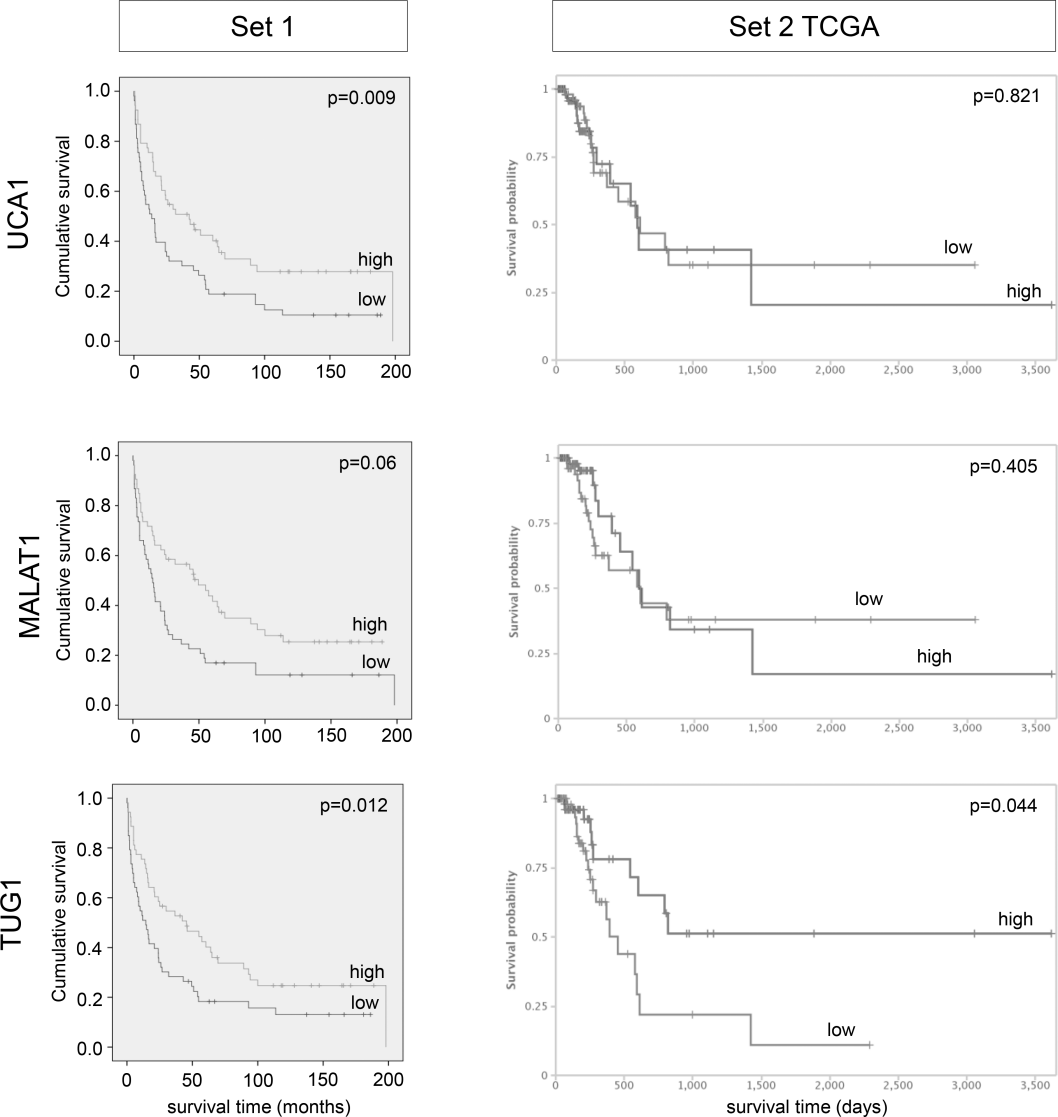
Impact of lncRNA expression levels on patient overall survival in tissue set 1 and 2. Kaplan-Meier curves are shown for lncRNA candidates displaying significant association with overall survival in set 1 (stratified by median expression; p-values for Cox regression analysis, time in months). Kaplan-Meier curves for set 2 were obtained from the TANRIC database (time in days).

In the univariate analysis patients with high *UCA1* expression from tissue set 1 (above median) had a considerably better overall survival (HR 0.567; 95% CI, 0.367–0.876; p = 0.011; Table 2), which also remained significant in both multivariate analysis (p = 0.015; Table 3) and Kaplan-Meier survival curve (Fig 2, left). Concurring results, albeit with lower significance levels, were obtained in both uni-and multivariate analyses of muscle-invasive tumours only (S3 Fig, S4 and S5 Tables). In the TCGA dataset, neither high nor low expression of *UCA1* was associated with clinical outcome.

**Table 2:** Univariate analyses of the impact of lncRNA expression on patient survival.

Variables	Overall survival			Disease-specific survival		
	HR	95% CI	P	HR	95% CI	P
**Age**						
**≤ 65**	ref.			ref.		
**> 65**	1.015	0.659–1.562	0.948	0.929	0.563–1.533	0.774
**Sex**						
**Female**	ref.			ref.		
**Male**	0.650	0.410–1.030	0.067	0.655	0.384–1.117	0.121
**Stage**						
**Ta-T1**	ref.			ref.		
**T2-T3**	2.187	1.277–3.742	**0.004**	5.167	2.219-12-034	**<0.001**
**Grade**						
**G1-G2**	ref.			ref.		
**G3**	2.458	1.512–3.996	**<0.001**	4.091	2.125–7.877	**<0.001**
**Lymph node status**						
**pNx / N0**	ref.			ref.		
**pN+**	3.551	2.161–5.837	**<0.001**	5.106	2.952–8.832	**<0.001**
**UCA1 exp. 50%**						
**low**	ref.			ref.		
**high**	0.567	0.367–0.876	**0.011**	0.657	0.398–1.084	0.100
**Linc-UBC1 exp. 50%**						
**low**	ref.			ref.		
**high**	0.953	0.619–1.468	0.828	0.966	0.587–1.592	0.893
**TUG1 exp 50%**						
**low**	ref.			ref.		
**high**	0.579	0.375–0.895	**0.014**	0.588	0.356–0.972	**0.038**
**ncRAN exp. 50%**						
**low**	ref.			ref.		
**high**	0.416	0.543–1.287	0.836	0.605	0.365–1.003	0.052
**MALAT1 exp. 50%**						
**low**	ref.			ref.		
**high**	0.547	0.353–0.848	**0.007**	0.591	0.357–0.78	**0.041**
**H19 exp. 50%**						
**low**	ref.			ref.		
**high**	0.904	0.587–1.390	0.644	0.925	0.562–1.523	0.761
**GAS5 exp. 50%**						
**low**	ref.			ref.		
**high**	0.707	0.459–1.091	0.118	0.697	0.423–1.150	0.158

Hazard ratios (HR) with 95% confidence intervals (CI) and p-values (p) were calculated by Cox regression analyses on overall and disease-specific survival for lncRNA expression levels in set 1. Patients were divided into a low- and a high-expression group for each lncRNA by median expression. Bold printed p-values were significant (≤0.05).

**Table 3:** Multivariate analyses of the impact of lncRNA expression on patient survival.

		**Overall survival**		**Disease-specific survival**		
**Variables**	**HR**	**95% CI**	**p**	**HR**	**95% CI**	**p**
**All cases (Ta-T4)**						
**Stage (T2-T4)**	1.329	0.697–2.536	0.388	2.547	0.982–6.603	0.054
**Grade (G3)**	1.784	1.001–3.178	**0.050**	2.200	1.052–4.598	**0.036**
**Lymph node metastasis (N+)**	2.982	1.748–5.085	**<0.001**	3.652	2.050–6.504	**<0.001**
**UCA1 (> 50%)**	0.573	0.366–0.897	**0.015**	0.669	0.400–1.121	0.127
						
		**Overall survival**		**Disease-specific survival**		
**Variables**	**HR**	**95% CI**	**p**	**HR**	**95% CI**	**p**
**All cases (Ta-T4)**						
**Stage (T2-T4)**	1.217	0.646–2.296	0.543	2.412	0.941–6.183	0.067
**Grade (G3)**	1.734	0.969–3.104	0.064	2.119	1.008–4.456	**0.048**
**Lymph node metastasis (N+)**	3.196	1.883–5.427	**<0.001**	3.865	2.176–6.857	**<0.001**
**TUG1 (> 50%)**	0.616	0.392–0.967	**0.035**	0.656	0.390–1.103	0.112
						
		**Overall survival**		**Disease-specific survival**		
**Variables**	**HR**	**95% CI**	**p**	**HR**	**95% CI**	**p**
**All cases (Ta-T4)**						
**Stage (T2-T4)**	1.156	0.604–2.211	0.662	2.199	0.843–5.738	0.107
**Grade (G3)**	2.007	1.126–3.575	**0.018**	2.264	1.075–4.768	**0.032**
**Lymph node metastasis (N+)**	3.017	1.778–5.120	**<0.001**	3.784	2.129–6.724	**<0.001**
**ncRAN (> 50%)**	1.003	0.637–1.580	0.991	0.789	0.466–1.334	0.376
						
		**Overall survival**		**Disease-specific survival**		
**Variables**	**HR**	**95% CI**	**p**	**HR**	**95% CI**	**p**
**All cases (Ta-T4)**						
**Stage (T2-T4)**	1.073	0.564–2.044	0.830	2.206	0.852–5.713	0.103
**Grade (G3)**	1.954	1.108–3.448	**0.021**	2.365	1.141–4.899	**0.021**
**Lymph node metastasis (N+)**	2.963	1.748–5.023	**<0.001**	3.608	2.038–6.388	**<0.001**
**MALAT1 (> 50%)**	0.625	0.400–0.976	**0.039**	0.765	0.459–1.276	0.304

*UCA1*, *TUG1*, *ncRAN* and *MALAT1* expression were analysed in multivariate analyses including tumour stage, grade and lymph node metastasis as parameters. Hazard ratios (HR) with 95% confidence intervals (CI) and p-values (p) are given for overall and disease-specific survival. Bold printed p-values are significant (≤0.05).

The major isoform of *MALAT1* has no intronic sequences, but many different splice variants are annotated in the *ensembl* database. Therefore, we first compared results for *MALAT1* expression in UC cell lines obtained with primer pairs covering splice variants in the 5’-region or 3’-region. Expression measured by both primer pairs correlated very well, revealing only two UC cell lines with high expression compared to benign cells (S2 Fig). Thus, we performed RT-qPCR analyses for the tissue samples only with the primer pair for the 3’-region, which has also been applied in reports by Diederichs and colleagues who have studied MALAT1 and other lncRNAs extensively [24]. The median expression of *MALAT1* tended to be lower in cancer tissues compared to normal controls in both sample sets, but the differences were not significant (Fig 1). However, comparing *MALAT1* expression between NMIBC and MIBC revealed that reduced lncRNA expression in UC originated mainly from muscle-invasive tumours; this effect was even significant in our own sample set 1 (p = 0.05; Table 1; S3 Fig left). These findings do not fit with previous reports that *MALAT1* expression is increased in UC and associated with distant metastasis [25,26]. In fact, our uni- and multivariate analyses revealed a poor prognosis for patients with reduced *MALAT1* expression (set 1 univariate Table 2: HR 0.547, 95% CI, 0.353–0.848, p = 0.007; set 1 multivariate Table 3: HR 0.625, 95% CI, 0.40–0.976, p = 0.039; TCGA set 2 univariate S6 Table: HR 0.67, 95% CI, 0.558–0.99, p = 0.044). Kaplan-Meier analysis suggested a shorter overall survival time for patients with low *MALAT1* expression (p = 0.06), especially in the lowest quartile (Fig 2 and S4 Fig).

For the lncRNA *H19* no significant expression changes overall were observed in either patient cohort (Fig 1), although individual NMIBC or MIBC tumour samples showed a clear upregulation. Similarly, only two of 12 UC cell lines displayed highly increased expression compared to benign cells (S2 Fig).

Likewise, *GAS5* expression was not significantly altered in both cohorts (Fig 1). In all UC cell lines, expression was reduced compared to the normal control (S2 Fig).

A long and a short isoform of *ncRAN* have been described [10]. Expression analysis by RT-qPCR in tumour cell lines revealed a strong correlation between both isoforms (S2 Fig). Therefore, we chose to measure the long isoform in the tissue sets. Concordantly between datasets 1 and 2, median expression of *ncRAN* was clearly elevated in tumour tissues by approximately 1.7-fold, but the difference was only statistically significant in set 2 (p<0.001, Fig 1). Accordingly, increased *ncRAN* expression was associated with overall survival of TCGA patients in univariate analysis (HR 1.53, 95% CI, 1.036–2.249, p = 0.032, S6 Table), but not in multivariate analysis. However, expression was neither significantly associated with stage, grade nor outcome in set 1 (Tables 1 and 2, S3 Fig, S4 Table).

Several tumour tissues analysed by RT-qPCR in set 1 clearly overexpressed *linc-UBC1*, but overall median expression was only slightly augmented and the difference between tumour and benign tissues was accordingly not significant (Fig 1). Generally, linc-UBC1 expression was very low, in four UC cell lines too, expression was below the detection limit (S2 Fig). Inconsistent with a previous report [11], no significant association was observed with lymph node metastasis or survival (Table 1, S5 Fig). Instead, *linc-UBC1* was much stronger expressed (4-fold in the median) in low grade compared to high grade tumour tissues. In set 2, *linc-UBC1* was significantly overexpressed (p<0.001, Fig 1), correlating with worse survival (p = 0.028, S5 Fig).

While we observed neither significant differential expression of *TUG1* in UC cell lines (S2 Fig), nor in sample set 1 (Fig 1 left),nor a significant correlation between *TUG1* expression and either tumour grade or stage (S3 Fig, Tables 1 and 2), *TUG1* was significantly increased in the set 2 TCGA cohort (p = 0.001, Fig 1 right). Intriguingly, *TUG1* was the only candidate that displayed significant results for both data sets in Kaplan-Meier survival analyses (Fig 2). Surprisingly, despite a general tendency towards overexpression, Kaplan-Meier analysis revealed that in both sample sets a subgroup of patients with low *TUG1* expression faced a poor prognosis with worse overall survival (Fig 2, set 1 p = 0.012; set 2 p = 0.044). This association between low *TUG1* expression and poor overall survival remained significant for uni- and multivariate analysis of set 1 (univariate Table 2: HR 0.579, 95% CI, 0.375–0.895, p = 0.014; multivariate Table 3: HR 0.616, 95% CI, 0.392–0.967, p = 0.035).

### Differential expression of lncRNA candidates among molecular subtypes of UC

**Fig 3:**
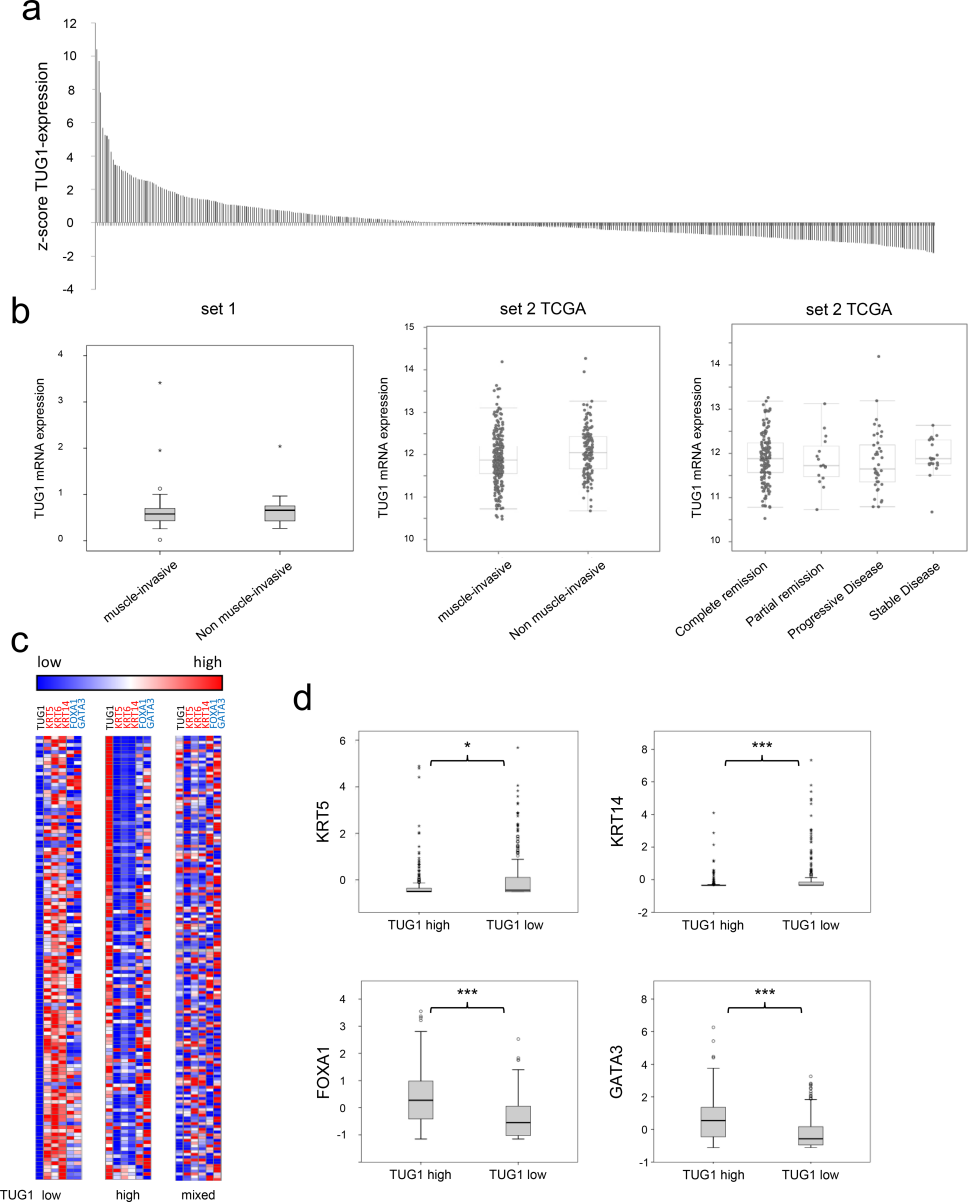
Correlation between low *TUG1* expression in UC and Basal-Squamous-like subtype. (a) Waterfall plot representing *TUG1* expression in tumour samples of the extended TCGA tumor tissue set (n = 408). *TUG1* expression is given as z-scores. The reference for calculating z-scores of RSEM data in TCGA studies are diploid samples. Samples with high expression had a z-score above 0, specimen with low expression had negative values. (b) Boxplot representations comparing *TUG1* expression in muscle-invasive and non muscle-invasive tumours of set 1 and extended set 2. (c) Heat map clustering for *KRT5*, *KRT6*, *KRT14*, *FOXA1* and *GATA3* with *TUG1* expression in the TCGA cohort (expression levels are given as a colour gradient between dark blue (low expression) and red (high expression)). The tumour cluster with low *TUG1* expression is shown in the left panel, that with a high expression in the centre panel and tumours with an intermediate expression pattern cluster in the right panel. (d) Expression levels of *KRT5*, *KRT14*, *FOXA1* and *GATA3* in tumours from the TCGA cohort with a *TUG1* expression above median (*TUG1* high) and below median (*TUG1* low). P-values for difference between high and low *TUG1* expressing group were calculated by Mann-Whitney U-test (*p≤0.05, ***p≤0.001).

To further elucidate the association between low *TUG1* expression and poor prognosis in a subgroup of patients, we investigated an extended set of TCGA tumour samples (provisional set, n = 408) that also contains a higher number of non-muscle-invasive cases. As illustrated in Fig 3A, *TUG1* was again highly expressed in a subset of tumours (n = 166, 40.7%), but 59.3% of tumour samples (n = 242) displayed low TUG1 expression. As in set 1 (Fig 3B left), in the extended TCGA dataset more cases with low TUG1 expression were found among the muscle-invasive tumours (Fig 3B right).

**Fig 4:**
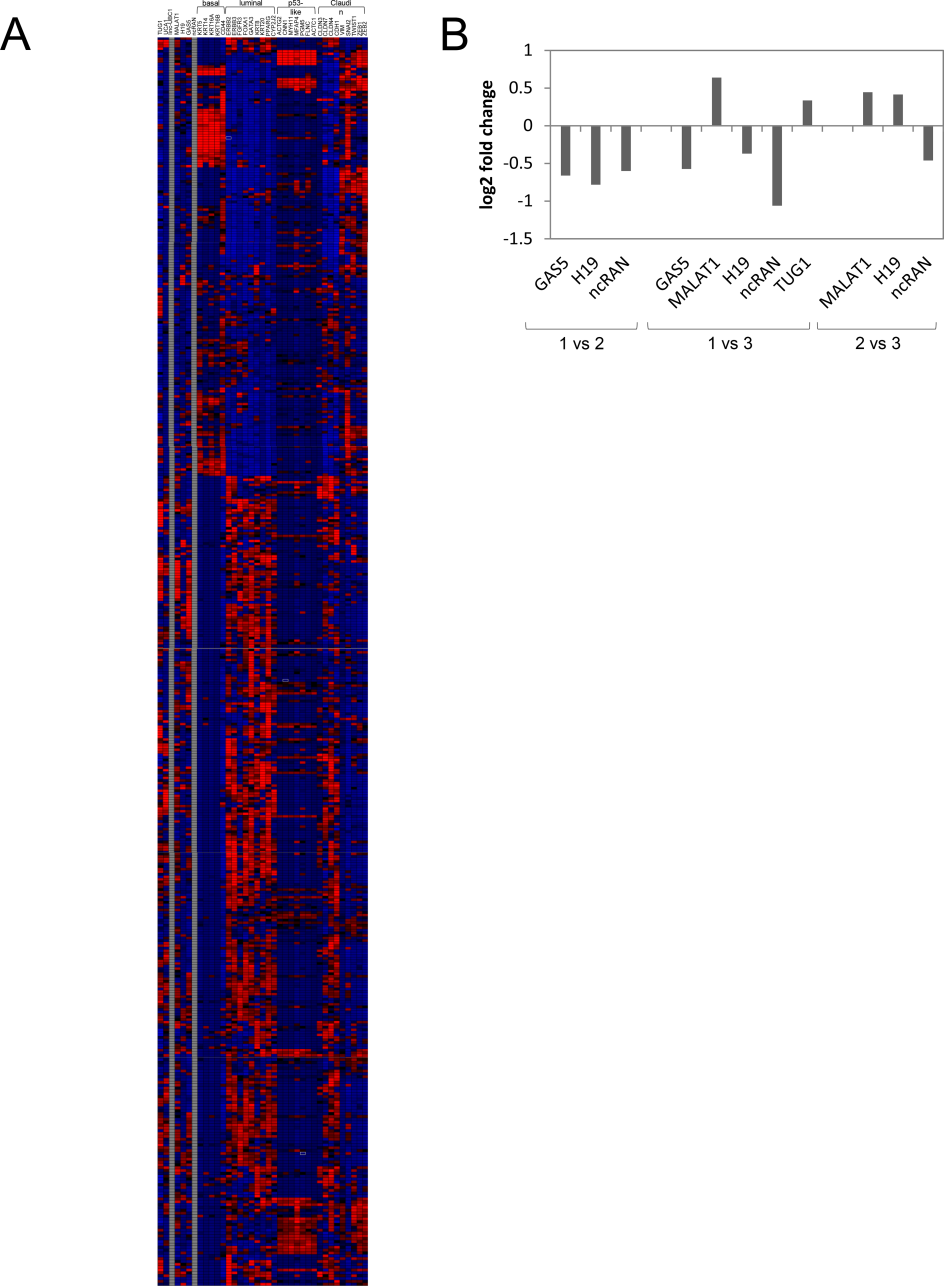
Association of differential lncRNA expression with molecular subtypes of UC. (a) TCGA expression data (z-scores) of the extended dataset (n = 408) was downloaded for genes defining molecular subtypes of MIBC according to Dadhania et al [28] and samples were assigned to the indicated subtypes by hierarchical clustering analysis using Genesis 1.0. Expression data of lncRNA candidates was downloaded accordingly, data was not available for *linc-UBC1* and *ncRAN*. (b) Illustration of differential expression (log2 fold change) of lncRNA candidates between molecular subclasses of NMIBC based on data published by Hedegaard et al (see reference for S3 Table) [29].

We then investigated whether *TUG1* expression might relate to any molecular subtype of UC. While the discussion about the number of different molecular subtypes in UC and their according gene signatures is ongoing, a consensus has been reached on the existence of basal-squamous-like subtype (BASQ) [27], which is consistently associated with poor prognosis across all studies. According to the consensus its minimal subset of expression markers includes increased expression of cytokeratins (*KRT) 5*, *6* and *14* and reduced expression of *FOXA1* and *GATA3*. Indeed, in the extended TCGA data set most tumours with low *TUG1* could be assigned to the BASQ type with significant upregulated *KRT5 and KRT14* expression and significantly lower expression of luminal markers *FOXA1* and *GATA3* (Fig 3C and 3D, Fig 4A). On the contrary, tumours with significantly increased *TUG1* expression, which encompass 8% of TCGA cases, were usually positive for markers of a luminal subtype, e.g. *FOXA1*, *GATA3*, but not for basal *KRTs* (Fig 3C). Notably, about one third of cases with variable TUG1 expression could not be straightforwardly assigned to a molecular subtype (intermediate pattern).

To substantiate the association of low *TUG1* expression with the BASQ subtype, we included further marker genes reported to define molecular subtypes of UC. Since no definitive consensus signatures for the other molecular subtypes have been agreed on, we applied a marker signature published in a recent meta-analysis by Dadhania et al [28]. Hierarchical clustering analysis of the extended TCGA data set for a panel of 30 coding genes distinguishing between subtypes and the lncRNA candidates demonstrated again an association between low *TUG1* expression and the basal-like subtype (Fig 4A). Similar expression patterns across these tumour samples became evident for *UCA1* and *MALAT1* (Fig 4A), suggesting that patients with low expression of these lncRNAs might also belong to the BASQ subtype.

A recent large-scale study on NMIBC by Hedegaard and colleagues reported that NMIBC can be subgrouped into at least three molecular subclasses [29]. This study compared tumour samples among each other and not differential expression compared to benign tissues, which are not usually available from these patients. Therefore, we could not simply use this data to confirm our results on differential expression between NMIBC, MIBC and benign tissues. However, some of our lncRNA biomarker candidates were also found to be differentially expressed among subclasses of NMIBC. *GAS5*, *H19* and *ncRAN* displayed the highest expression levels in differentiated class 1 tumours, *MALAT1* and *TUG1* were rather highly expressed in basal-like class 3 tumours (Fig 4B, see S3 Table in Ref. 29). Taken together, these data suggest that lncRNA expression may not only vary between NMIBC vs MIBC, but even between their respective molecular subtypes.

## Discussion

The major aim of this study was to validate the diagnostic and prognostic value of lncRNAs that were postulated in original publications and reviews as individual biomarkers for urothelial carcinoma [4,5,6,7,8,9,10,11,30]. With the exception of *GAS5*, all other candidates were reported to be significantly upregulated.

While urinary markers are evidently clinically useful, our study focused on tumour tissues based on the postulates that a robust and clinically applicable RNA biomarker for the identification of UC should be strongly and regularly overexpressed across large and independent cohorts of patients with UC and that its degree of overexpression should correlate with histopathological parameters indicating worse prognosis or independently predict prognosis. To this end, we analysed two distinct lncRNA expression datasets from different populations and obtained by different assays. Our own tissues sample set investigated by RT-qPCR came from an urban population in an industrial region of Germany (set 1), whereas the TCGA dataset (set 2) was assembled from US-American patients using next generation RNA-sequencing. Briefly, our analysis of public TCGA data for seven lncRNA candidates by means of the TANRIC database tool identified four of them to be significantly higher expressed in tumour tissues compared to control tissues, but for all but one the differences were slight, with medians differing by a factor less than 2-fold. In set 1, the direction of the expression changes between tumour and benign tissues concurred for all seven candidates with the TCGA data, but none of the candidate lncRNAs showed significant overexpression in tumours or reproducible strong prognostic value. Strikingly, for two candidates we obtained results from both datasets that were opposed to previous reports. *MALAT1* was reported to be overexpressed in UC [6], whereas we observed reduced expression in both sets. Conversely, *GAS5* was expected to be downregulated in UC [8], but was rather upregulated in both our cohorts.

There are many potential confounders that might account for the differences between the present study and previous reports, including technical issues like the cellularity of samples, quality of RNA and of the assays used as well as differences relating to patient populations. A closer analysis, as detailed below for the individual lncRNAs, suggests however that the most important factors relate to the biological heterogeneity of UC. Specifically, it is well known that non muscle-invasive tumours (NMIBC) differ substantially from muscle-invasive tumours (MIBC) with regard to tumour biology and molecular alterations. Thus, divergent results from different studies might originate from the varying abundance of tumour stages across the investigated cohorts. Most previous reports have investigated mixed cohorts of NMIBC and MIBC, whereas especially the TCGA cohort is heavily biased towards MIBC. However, expression differed significantly between NMIBC and MIBC only for *UCA1* and *MALAT1* and only in either set 1 or set 2. More prominently, our own results for MIBC along with a recent comprehensive transcriptome analysis of NMIBC by Hedegaard et al. [29] suggest a high degree of heterogeneity in the expression of lncRNAs between molecular subtypes of UC. Specifically, these authors reported that *GAS5*, *H19* and *ncRAN* were more strongly expressed in class 1 NMIBC, representing a more differentiated subtype with a lower risk for progression, whereas *MALAT1* and *TUG1* were more strongly expressed in the newly defined basal-like class 3 NMIBC [29]. Notably, in accord with this idea, lncRNAs have been shown to often exhibit strong cell-type and tissue-specific patterns of expression [2]. Therefore, it could be rather difficult to identify a single lncRNA biomarker that robustly distinguishes all stages and subtypes of UC in the same fashion as certain lncRNAs like *PCA3* are able to identify almost all prostate cancer cases [31]. Rather, lncRNAs like *TUG1* might turn out to be most valuable as biomarkers as part of a panel to cover UC subtypes for diagnosis.

The best studied lncRNA candidate to date is arguably *UCA1* which has been described as a potential urinary biomarker for UC by several groups. Notably, most of these studies have investigated urine sediments [32] rather than tissue samples, applying RT-qPCR [5] or semi-quantitative RT-PCR [33,34]. *UCA1* was originally reported to be largely restricted to embryonic tissues and cancers and not to be expressed in normal bladder tissue [34], but it is clearly detectable by sensitive techniques in benign bladder tissues. In UC tumour tissues, we found a moderate increase of *UCA1* expression, which in the TCGA set (set 2) resulted mainly from high expression levels in papillary tumours. Concurringly, a follow-up study on *UCA1* as a urinary biomarker found *UCA1* urine analysis to be particularly efficient for detection of pT1 tumours [33]. Conceivably, increased detection of *UCA1* in urinary sediments might reflect only partly increased expression in cancer tissues, but instead increased shedding of urothelial cells into urine in tumour patients. Analogously, *Cytokeratin 20* mRNA, a marker of differentiated urothelial cells expressed in many urothelial carcinomas, is increased in urinary sediments of UC patients [35].

As for *UCA1*, we observed overexpression of *linc-UBC1* in many UC, but significant increases only in the TCGA dataset. This finding fits reasonably with a previous report, whereby *linc-UBC1* was upregulated in 60% of 102 bladder cancer tissues by more than 1.5-fold [11]. Notably, *linc-UBC1* expression was very low in many tissues. Concordant with the previous report, high *linc-UBC1* expression was associated with worse survival in the TCGA cohort, but neither associated with survival nor lymph node status in the samples analysed by RT-qPCR. However, expression near the detection limit in many cancer tissues would limit assay sensitivity and thus suitability of *linc-UBC1* as a biomarker.

Unexpectedly, in view of previous reports on *MALAT1* overexpression in UC [2, 26], *MALAT1* expression was rather diminished in UC cell lines and tumour tissues compared to normal tissues in our cohorts. This discrepancy may relate to significant differences in *MALAT1* expression between NMIBC and MIBC. In our sample set 1 *MALAT1* expression was diminished in invasive tumours compared to benign tissues, whereas NMIBC displayed rather upregulation. Low *MALAT1* expression in MIBC, moreover, appears to be associated with the BASQ subtype, as revealed by hierarchical clustering analysis (Fig 4). The association of low *MALAT1* expression with poor survival may therefore reflect its association with the more clinically aggressive basal-like subtype. This contention is partly compatible with other reports in the literature. In a study by Fan et al. [25] 62 of 95 investigated bladder cancers were NMIBC and *MALAT1* was rather upregulated. In the large-scale transcriptome analysis of early-stage UC by Hedegaard et al [29] *MALAT1* was likewise expressed most prominently in one NMIBC subgroup, basal-like class 3. Thus, opposite to the results for muscle-invasive UC where low *MALAT1* expression was associated with basal-like characteristics, in NMIBC *MALAT1* upregulation correlated with basal-like characteristics, but not necessarily with disease progression. Additional published studies on *MALAT1* in UC are difficult to compare as only small sample numbers were investigated and information on histopathological characteristics is not comprehensive [26]. Two meta-analyses of 10 studies on *MALAT1* in various cancer types indicated a relatively consistent association of increased *MALAT1* expression with survival in various types of cancer, but some studies analysed only few cases [36, 37]. Clearly, further studies on *MALAT1* should be conducted to ascertain the association of its expression with tumour subtypes. Moreover, given the assumption that lncRNAs commonly act in a context-dependent fashion, the function of MALAT1 should be investigated in different UC molecular subtypes.

*H19* expression was not significantly altered between tumour and benign tissues of both sample sets and no association with tumour stage or other clinical parameters was found. Notably, *H19* expression varied across a broad range. *H19* is one of the first discovered long noncoding RNAs and its gene is imprinted. Altered expression could therefore be due to epigenetic as well as genetic changes of the imprinted gene cluster at 11p15.5. Previously, Luo et al. reported a strong increase in *H19* expression in 24 bladder tumour specimens over adjacent normal tissue [8], but strangely, expression measured by RT-qPCR ranged across 15 orders of magnitude. More recently, Li et al. [38] reported more moderate increases in 40 tumours (mostly NMIBC) vs. 19 benign tissues and an excellent association with grading. Hedegaard et al observed significant expression changes in their large NMIBC cohort and found *H19* the highest expressed lncRNA in well-differentiated class 1 tumours with a low risk for progression [29]. In an older report, *H19* mRNA expression in bladder cancer was studied by in situ hybridization on paraffin sections [39]. Thus, again, varying tumour population and detection techniques might account for the differences among the findings.

For *GAS5*, we observed neither significant differential expression between tumour and benign tissues nor associations of expression with histopathological parameters or prognosis. However, expression was increased in many NMIBC tumours compared with benign tissues. Accordingly, Hedegaard et al [29] observed significantly increased *GAS5* expression in class 1 NMIBC tumours. Previously, *GAS5* had been reported to be downregulated based on a cohort of 12 NMIBC and 16 MIBC [8], but the differences between these subtypes were not detailed. Based on these results and experiments in one bladder cancer cell line, a tumour suppressive function was postulated [40], which would not fit well with the newer results in tissues.

For *ncRAN*, deregulation has been reported in neuroblastoma [41], colorectal cancer [42] and in bladder cancer, where a limited number of cancer tissues were investigated by semi-quantitative PCR [10]. Experimental overexpression of *ncRAN* enhanced cell proliferation, migration and invasion in the well-differentiated UC cell line RT-4. Accordingly, *ncRAN* tended to be overexpressed, albeit moderately, in the cancer tissues in our study, but the association of lower expression with worse prognosis in one sample set argues against an important function of *ncRAN* upregulation in tumour progression. In addition, Hedegaard et al reported increased *ncRAN* expression in differentiated and good prognosis class 1 tumours likewise arguing against a decisive function in tumour progression [29].

Upregulation of *TUG1* and an association with higher tumour stages in UC was reported in previous studies. In the first study, *TUG1* was elevated by 1.74-fold in the mean in 44 cancer tissues compared to adjacent normal tissues [9]. Another study detected *TUG1* transcripts in 54 bladder cancer tissues by RT-qPCR; expression in normal tissues was not investigated [43]. A third study observed significant overexpression in 47 muscle-invasive tumours compared to paired benign tissues, an association with metastasis and shorter overall survival [44]. We found modest, but not consistently statistically significant increases of *TUG1* in cancer tissues across both tissue sets. Strikingly, *TUG1* was the only investigated lncRNA candidate that was significantly associated with survival in Kaplan-Meier analyses across both cohorts, but unexpectedly, low rather than high *TUG1* expression was significantly correlated with poor overall survival, which remained significant in multivariate analysis. Interestingly, Zhang et al. likewise found diminished *TUG1* expression in non-small cell lung carcinoma (NSCLC) to be associated with higher tumour stage and size as well as significant poorer overall survival in uni- and multivariate analyses [45]. In their study, *TUG1* knockdown in NSCLC cell lines promoted cellular proliferation, whereas in T24 bladder cancer cells *TUG1* overexpression increased cell invasion [9, 44].

A new finding in our study was the association of low *TUG1* expression with a particular molecular subtype of muscle-invasive UC, the basal subtype with squamous differentiation patterns (BASQ). While the definition of molecular subtypes in UC is still somewhat controversial, a consensus has been reached on the existence of this particular subtype among MIBC, its association with poor prognosis and a minimal set of defining markers [27]. Among five markers for BASQ, low *TUG1* expression significantly correlated with four, i.e. high expression of C*ytokeratins 5* and *14* and low expression of *FOXA1* and *GATA3*. The correlation of low *TUG1* expression with poor prognosis might therefore reflect its relation to the BASQ molecular subtype with its known worse clinical behaviour. As discussed above, low expression of *MALAT1* and *UCA1* might similarly be related to the BASQ subtype.

Taken together, these observations hint at cancer subtype-specific expression and context-dependent functions of *TUG1* which should be considered in future studies. Of note, Zhang et al. proposed *TUG1* as a direct transcriptional target of p53 and reported that *TUG1* was not induced in cell lines depleted of *p53* or harbouring common *p53* mutations [45]. *P53* mutations are common in urothelial carcinomas, especially in the BASQ subtype, and might explain low *TUG1* expression in some tumour tissues.

In conclusion, we observed that few of the previously reported changes in lncRNA expression or their association to histopathological parameters and patient prognosis could be robustly confirmed across two further large independent patient cohorts. In particular, overexpression of lncRNAs was often restricted to a subset of the cases or was moderate in extent. In addition to technical issues, varying proportions of tumour stages and molecular subtypes are likely to account for the differences between studies. Notably, both our sample set and the TCGA cohort consist predominantly of muscle-invasive tumours and our data are not representative for NMIBC. Moreover, the case of *UCA1* demonstrates that tissue and urinary biomarkers might differ. Taken together, our study clearly demonstrates that the identification of reliable lncRNA biomarkers for urothelial carcinoma demands validation studies in independent patient cohorts with large sample numbers which take tumour stages and molecular subtypes into account. In addition, lncRNAs might deserve investigation as components of biomarker panels for UC subtypes.

## Supporting information

S1 Fig Quality controls for tissues of set 1. (a) To further characterize the quality/ purity of tissue samples from set 1 expression of the proliferation marker Ki67 (gene: *MKI67*) was determined in all samples by RT-qPCR. RNA expression illustrated as boxplot representation (relative expression to geometric mean of reference genes *SDHA* and *TBP*). P-values for difference between benign (Normal) and tumour samples (Tumour) were calculated by Mann-Whitney U-test. (b) Two representative H&E stainings of tumour tissue sections used to evaluate histology, quality and cellularity of the sample are given.
(TIF)

S2 Fig lncRNA expression in UC cell lines. Relative expression of the seven lncRNA candidates across 12 UC cell lines and a benign control cell line (TERT-NHUC) is illustrated in bar graphs. Cell lines were classified into non-basal like and basal-like according to Earl et al [20]. Two different primer assays detecting various numbers of transcript variants had been evaluated for *MALAT1* and *ncRAN* across the cell lines.
(TIF)

S3 Fig lncRNA expression data of set 1 and 2 analysed separately for NMIBC and MIBC. Boxplot representations of lncRNA expression in set 1 (left, RT-qPCR, relative expression to geometric mean of reference genes SDHA and TBP) and set 2 (right, RNA-Seq in the TCGA bladder cancer cohort, expression as log2 RPMK, data obtained from the TANRIC database). P-values for difference between control (N) samples, non-muscle invasive stages (pTa/pT1, NMIBC) and muscle-invasive tumours (T2-T4, MIBC) were calculated by Mann-Whitney U-test.
(TIF)

S4 Fig Kaplan Meier Analysis for overall survival of patients of set 1 divided in subgroups based on lncRNA expression. Kaplan Meier analysis for quartiles of lncRNA expression in set 1 as indicated and overall survival (stratified by three cutpoints, time in months).
(TIF)

S5 Fig Impact of lncRNA expression levels on patient overall survival in tissue set 1 and 2. Kaplan-Meier curves are shown for each lncRNA stratified by median expression. Kaplan-Meier curves for set 2 were obtained from the TANRIC-database. Only data for lncRNAs which did not show a significant difference for set 1 are shown (p-values for Cox regression analysis).
(TIF)

S6 Fig Analysis of RT minus controls. RT minus controls without reverse transcriptase were included in the analysis of assays using primers that were not exon spanning to exclude that results were affected by contamination with genomic DNA. Raw quantified values are given (related to the internal standard curve for the respective gene). Without RT fluorescence signals remained below the detection limit (n.d.).
(TIF)

S1 Table Primer sequences and PCR conditions. Primer sequences used in RT-qPCR analyses in 5’->3’ orientation and further information regarding PCR assay design, reaction conditions and the reference bladder or prostate cancer cell line used for standard curve (Ref. std. curve) are given.
(PDF)

S2 Table Information on RT-qPCR run performance. For each lncRNA three RT-qPCR runs were conducted with normal and tumor samples distributed equally. Information on slope of the standard curve, resulting efficiency, R^2, melting temperature Tm, the Y-Intercept and Cq values of negative controls (Cq neg. “-”equivalent to undetectable) are given for each run. *According to the melting curve analysis the Cq result for the negative control did not result from a contamination by the specific amplicon.
(PDF)

S3 Table Information on RT-qPCR run performance (2nd). For the second reverse transcription of tissue sample RNA RT-qPCR runs were conducted with normal and tumor samples distributed equally. Information on slope of the standard curve, resulting efficiency, R^2, melting temperature Tm, the Y-Intercept and Cq values of negative controls (Cq neg. “-”equivalent to undetectable) are given for each run. *According to the melting curve analysis the Cq result for the negative control did not result from a contamination by the specific amplicon.
(PDF)

S4 Table Univariate analyses of the impact of lncRNA expression on survival of patients with MIBC. Hazard Ratios (HR) with a 95% Confidence Interval (CI) and p-values (P) were calculated by Cox regression analyses on overall and disease-specific survival for lncRNA expression levels for all patients with T2-T4 tumors in set 1. Patients were divided into a low- and a high-expression group for each lncRNA by median expression. The cut-off is based on the whole Ta-T4 cohort. Bold printed p-values were significant (≤0.05).
(PDF)

S5 Table Multivariate analyses of the impact of lncRNA expression on survival of patients with MIBC. UCA1, TUG1, ncRAN and MALAT1 expression had a statistically significant impact on patient overall or disease-specific survival and were further analysed in the multivariate analyses including tumor stage, grade and lymph node metastasis as parameters. Hazard Ratios (HR) with a 95% Confidence Interval (CI) and p-values (p) are given for overall and disease-specific survival. Bold printed p-values were significant (≤0.05).
(PDF)

S6 Table Univariate analyses of the impact of lncRNA expression on patient survival (TCGA). Hazard Ratios (HR) with a 95% Confidence Interval (CI) and p-values (P) were calculated by Cox regression analyses on overall and disease-specific survival for lncRNA expression levels in set 1. Patients were divided into a low- and a high-expression group for each lncRNA by median expression. Bold printed p-values were significant (≤0.05).
(PDF)

S1 File Supplementary methods. (PDF)
