# Supplementary figures and images for: Diagnostic and prognostic value of long noncoding RNAs as biomarkers in urothelial carcinoma
Source: PLoS One. 2017 Apr 21;12(4):e0176287. doi: 10.1371/journal.pone.0176287 (PMC5400278; doi:10.1371/journal.pone.0176287)

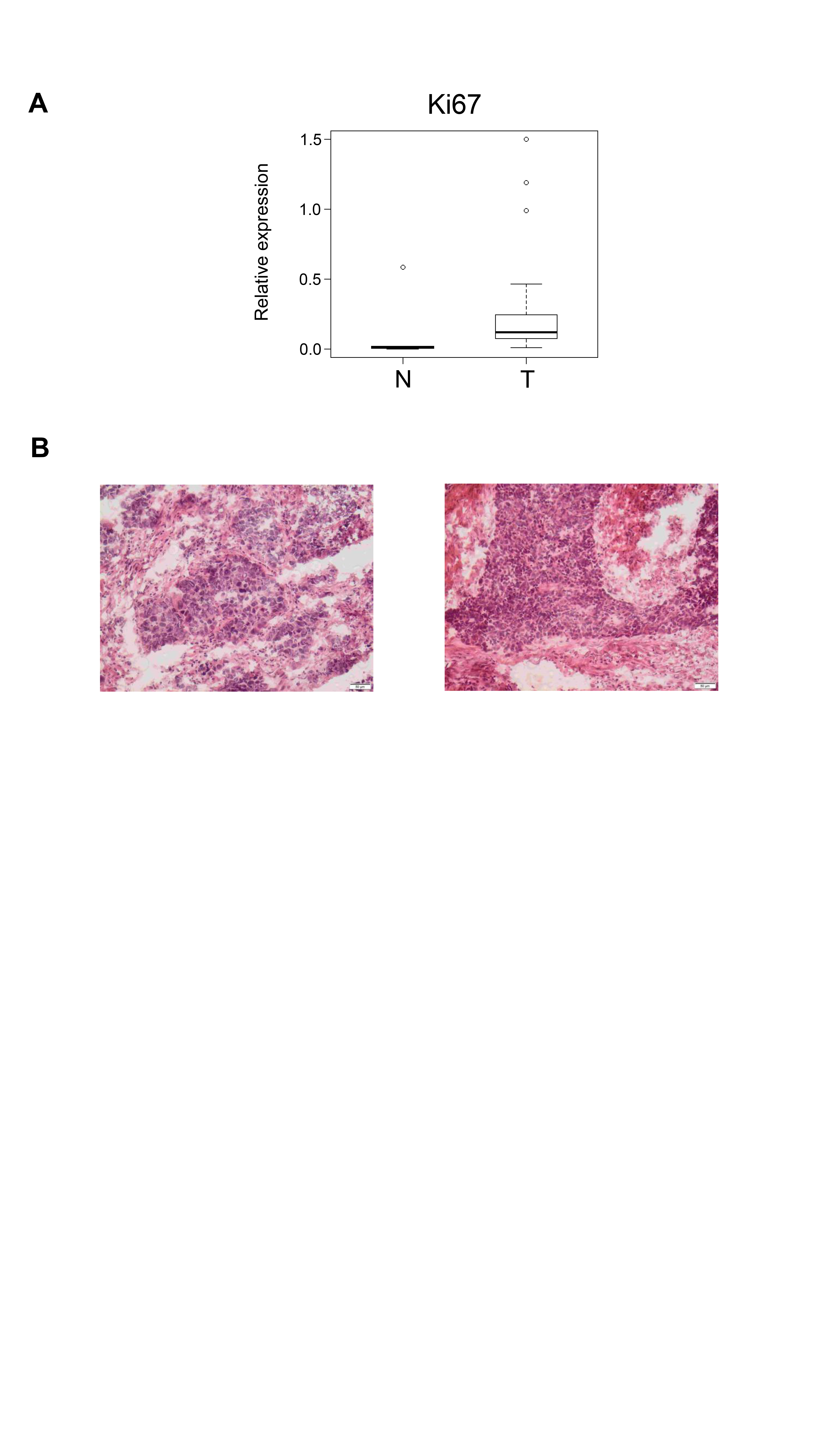

Supplement: S1 Fig — (a) To further characterize the quality/ purity of tissue samples from set 1 expression of the proliferation marker Ki67 (gene: MKI67) was determined in all samples by RT-qPCR. RNA expression illustrated as boxplot representation (relative expression to geometric mean of reference genes SDHA and TBP). P-values for difference between benign (Normal) and tumour samples (Tumour) were calculated by Mann-Whitney U-test. (b) Two representative H&E stainings of tumour tissue sections used to evaluate histology, quality and cellularity of the sample are given. (TIF) [file pone.0176287.s001.tif]

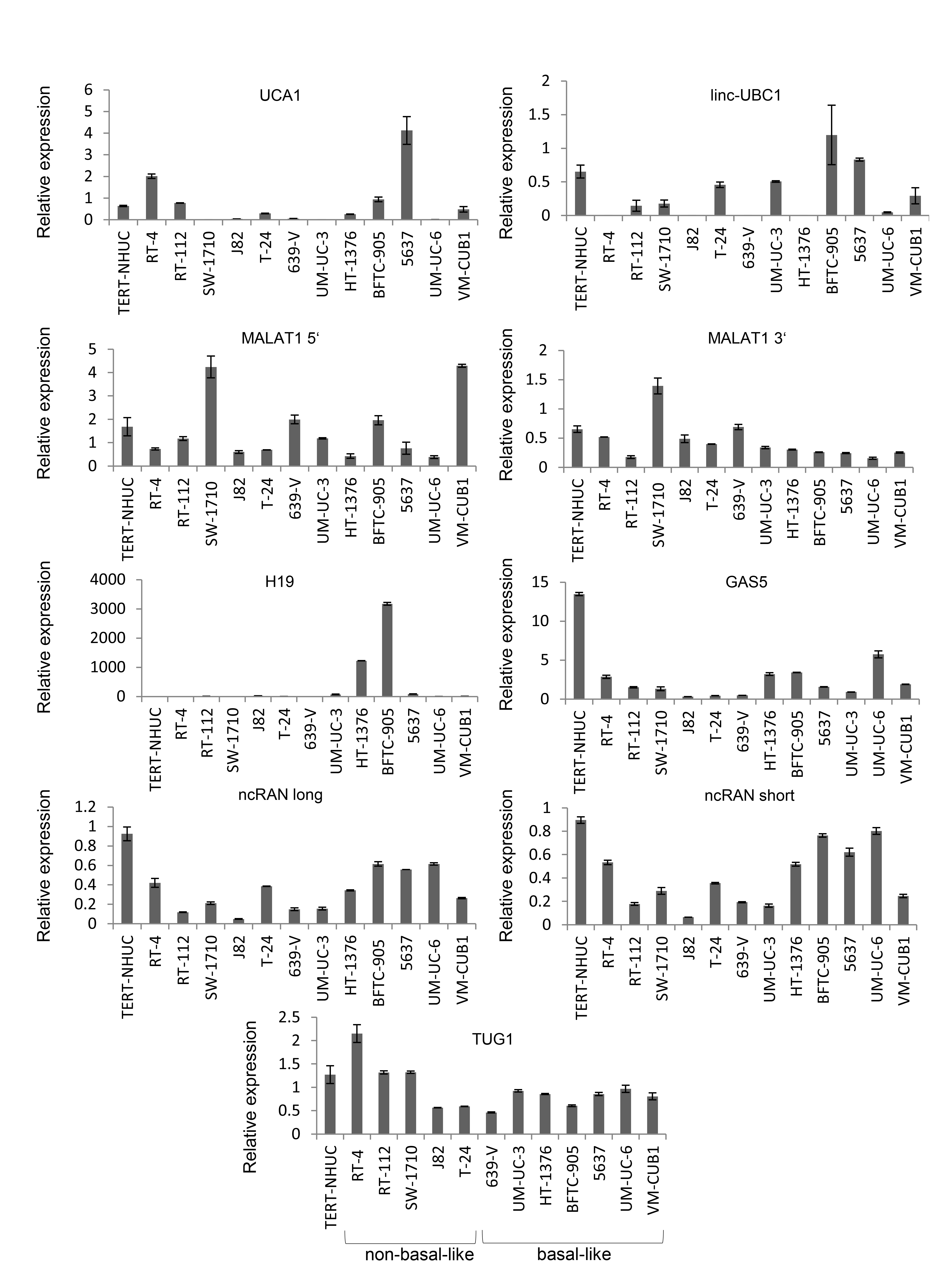

Supplement: S2 Fig — Relative expression of the seven lncRNA candidates across 12 UC cell lines and a benign control cell line (TERT-NHUC) is illustrated in bar graphs. Cell lines were classified into non-basal like and basal-like according to Earl et al [20]. Two different primer assays detecting various numbers of transcript variants had been evaluated for MALAT1 and ncRAN across the cell lines. (TIF) [file pone.0176287.s002.tif]

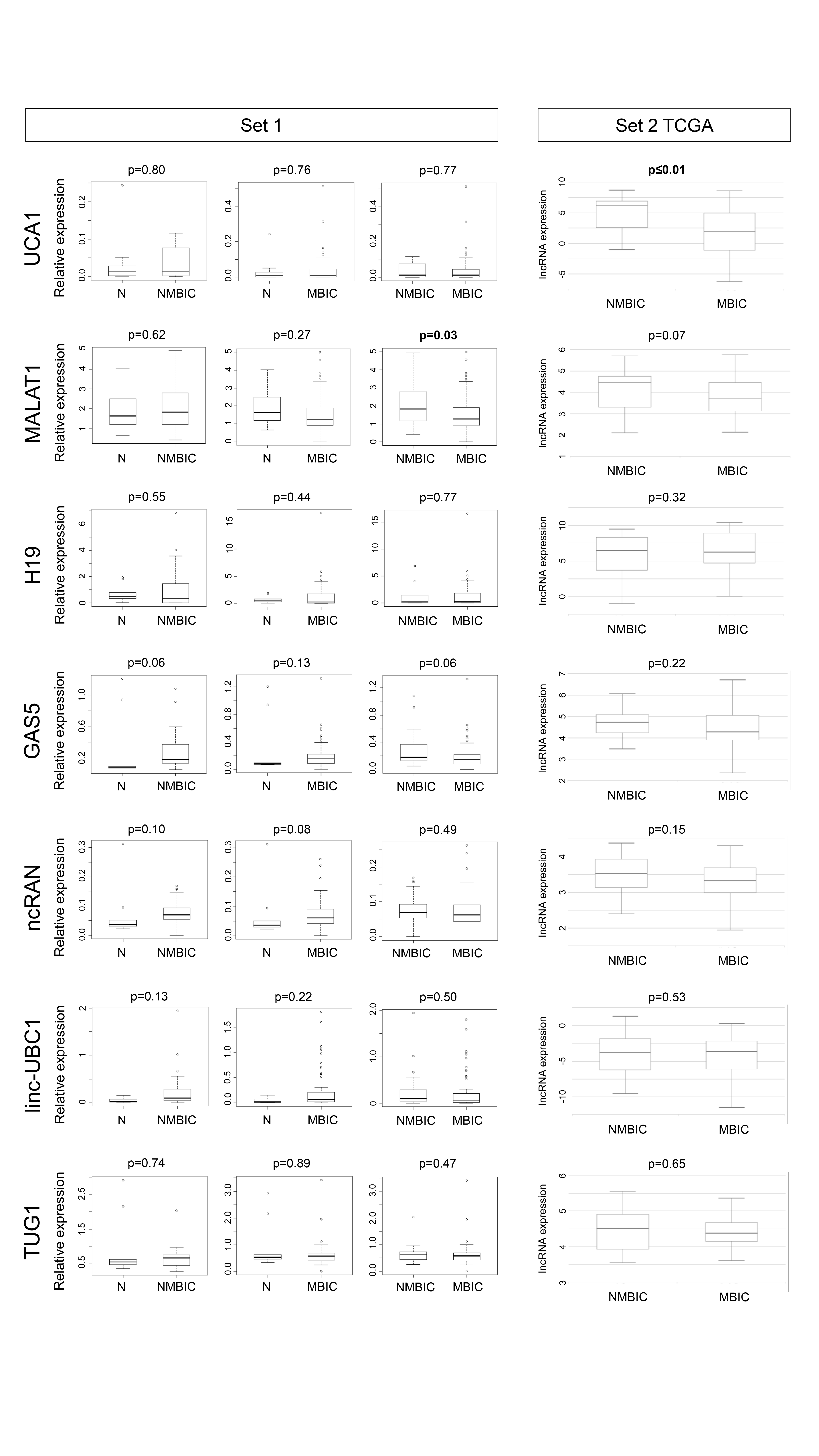

Supplement: S3 Fig — Boxplot representations of lncRNA expression in set 1 (left, RT-qPCR, relative expression to geometric mean of reference genes SDHA and TBP) and set 2 (right, RNA-Seq in the TCGA bladder cancer cohort, expression as log2 RPMK, data obtained from the TANRIC database). P-values for difference between control (N) samples, non-muscle invasive stages (pTa/pT1, NMIBC) and muscle-invasive tumours (T2-T4, MIBC) were calculated by Mann-Whitney U-test. (TIF) [file pone.0176287.s003.tif]

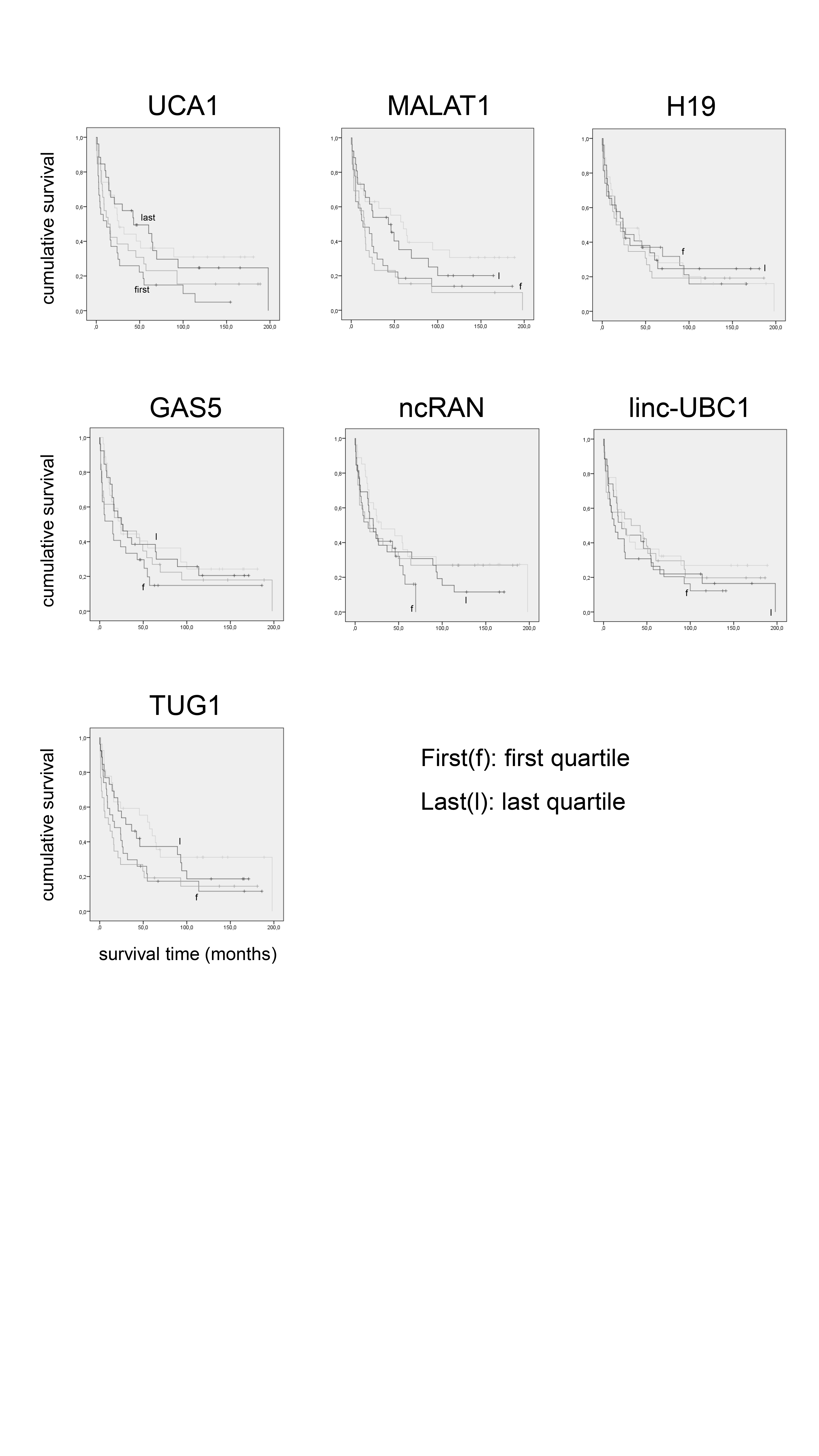

Supplement: S4 Fig — Kaplan Meier analysis for quartiles of lncRNA expression in set 1 as indicated and overall survival (stratified by three cutpoints, time in months). (TIF) [file pone.0176287.s004.tif]

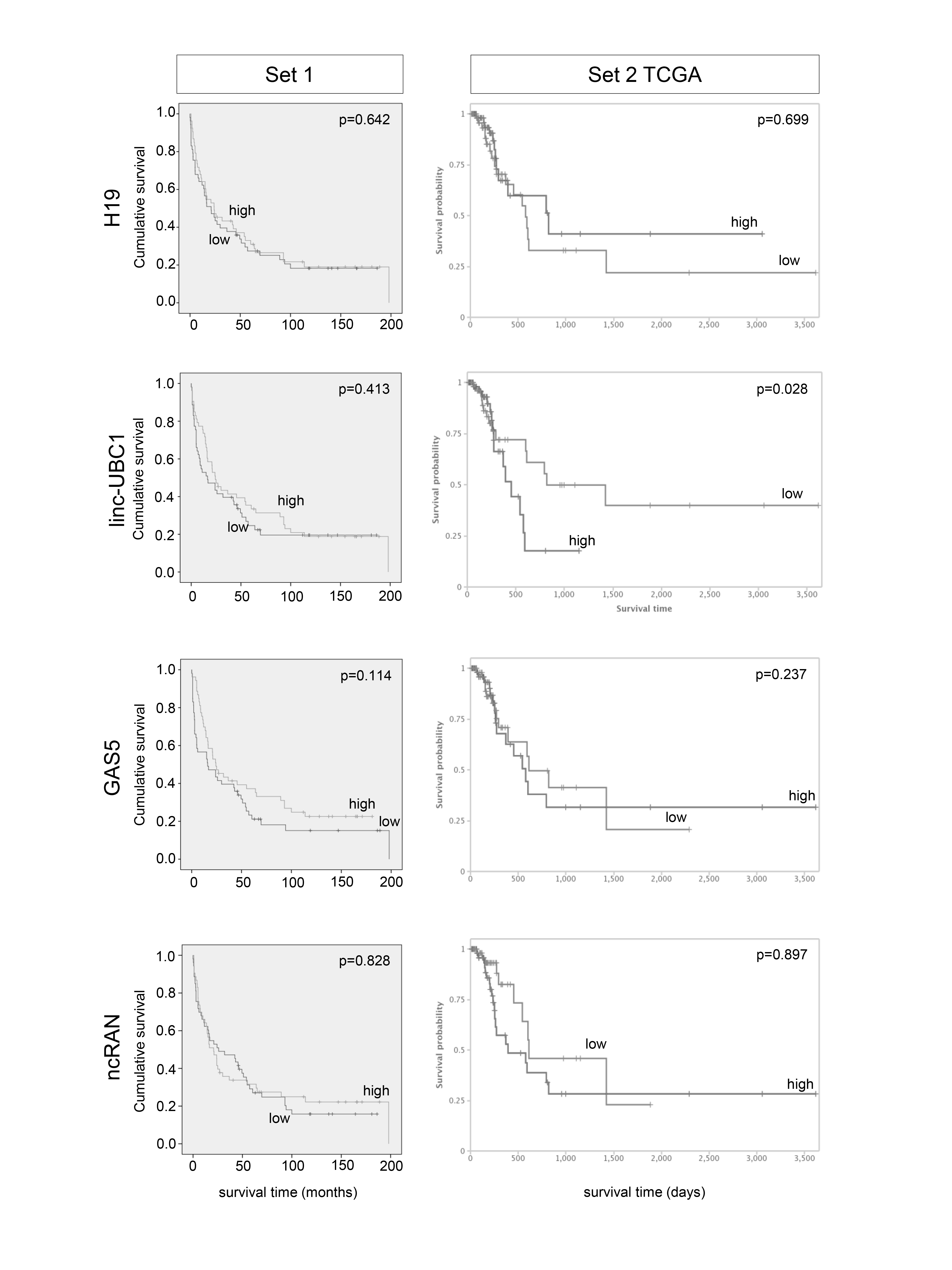

Supplement: S5 Fig — Kaplan-Meier curves are shown for each lncRNA stratified by median expression. Kaplan-Meier curves for set 2 were obtained from the TANRIC-database. Only data for lncRNAs which did not show a significant difference for set 1 are shown (p-values for Cox regression analysis). (TIF) [file pone.0176287.s005.tif]

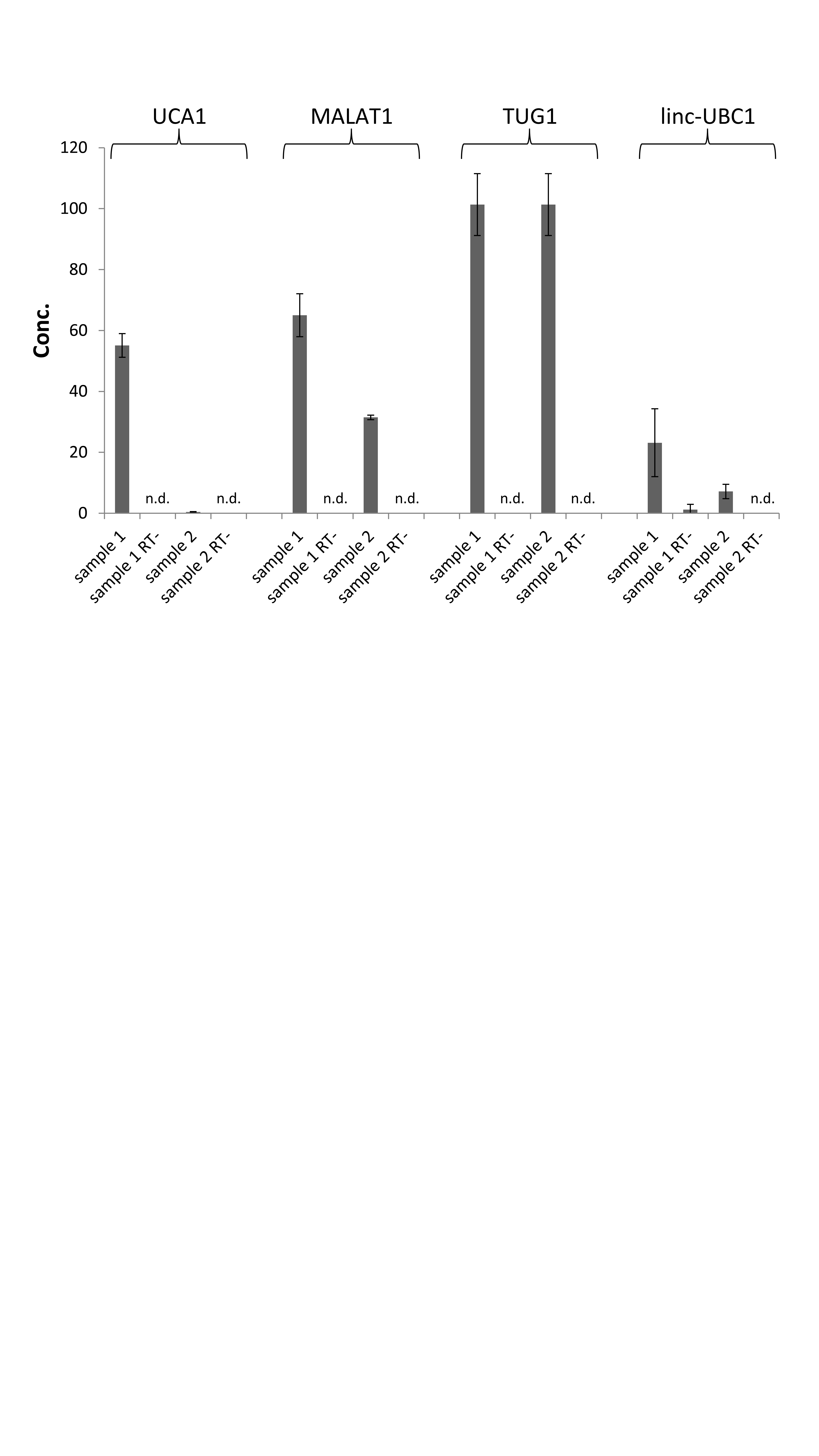

Supplement: S6 Fig — RT minus controls without reverse transcriptase were included in the analysis of assays using primers that were not exon spanning to exclude that results were affected by contamination with genomic DNA. Raw quantified values are given (related to the internal standard curve for the respective gene). Without RT fluorescence signals remained below the detection limit (n.d.). (TIF) [file pone.0176287.s006.tif]
