# Supplementary material for: Diagnostic and prognostic value of long noncoding RNAs as biomarkers in urothelial carcinoma
Source: PLoS One. 2017 Apr 21;12(4):e0176287. doi: 10.1371/journal.pone.0176287 (PMC5400278; doi:10.1371/journal.pone.0176287)
Supplement: S1 Table — Primer sequences used in RT-qPCR analyses in 5’->3’ orientation and further information regarding PCR assay design, reaction conditions and the reference bladder or prostate cancer cell line used for standard curve (Ref. std. curve) are given. (PDF) [file pone.0176287.s007.pdf]

Gene	Primer sequences	Annealing temp.	Ref. std. curve	amplicon length	localisation	Ensembl
**TBP**	Fwd: ACAACAGCCTGCCACCTTA<br>Rev: GAATAGGCTGTGGGGTCAGT	55°C	PC-3	120	Exon 2/3	ENSG00000112592
**SDHA**	Fwd: GCCAGGACCTAGAGTTTGTTCA<br>Rev: CTTTCGCCTTGACTGTTAATGA	55°C	RT-112	115	Exon7/8	ENSG00000073578
**UCA1**	Fwd: TTTTTTCCACACCCAAAACAA<br>Rev: ATTTCATGAGAGTAGGCTTGAGGA	55°C	5637	109	Exon3	ENSG00000214049
**MALAT1 5'**	Fwd: CTTCTGGGTGTGTCCCTGACTG<br>Rev: GCCATGGAAAGCGAGTTCAA	55°C	UM-UC-3	110	Exon1	ENSG00000251562
**MALAT1 3'**	Fwd: AAAGCAAGGTCTCCCCACAAG<br>Rev: GGTCTGTGCTAGATCAAAAGGCA	55°C	J-82	71	Exon1	ENSG00000251562
**TUG1**	Fwd: TTCCTACCACCTTACTACTGACG<br>Rev: GGAGGTAAAGGCCACATC	55°C	253J	93	Exon2	ENSG00000253352
**ncRAN_long**	Fwd: CAGTCAGCCTCAGTTTCCAA<br>Rev: AGGCAGGGCTGTGCTGAT	55°C	T-24	156	Exon2/3	ENSG00000163597
**ncRAN_short**	Fwd: ATGTTAGCTCCCAGCGATGC<br>Rev: CTAACTGCCAAAAGGTTT TCC	55°C	T-24	151	Exon2/3	ENSG00000163597
**linc-UBC1**	Fwd: CCTGCTTGGAAACTAATGACC<br>Rev: AGGCTCAACTTCCCAGACTCA	55°C	T-24	152	Exon1	ENSG00000281406
**GAS5**	Fwd: CTTGCCTGGACCAGCTTAAT<br>Rev: CAAGCCGACTCTCCATACCT	57°C	T-24	122	Exon6/7/8	ENSG00000234741
**H19**	Fwd: CACCAGCTGCCGAAGGCCAA<br>Rev: CCAGCCTAAGGTGTTCAGGAAGG	62°C	HT-1376	122	Exon2/3	ENSG00000130600
**Ki67**	Fwd: AGTGAAGGAGCAACCGCAGT<br>Rev: ATACACTGCCGTCTTAAGGGAG	57°C	T-24	219	Exon12/13	ENSG00000148773
