# Supplementary material for: Diagnostic and prognostic value of long noncoding RNAs as biomarkers in urothelial carcinoma
Source: PLoS One. 2017 Apr 21;12(4):e0176287. doi: 10.1371/journal.pone.0176287 (PMC5400278; doi:10.1371/journal.pone.0176287)
Supplement: S2 Table — For each lncRNA three RT-qPCR runs were conducted with normal and tumor samples distributed equally. Information on slope of the standard curve, resulting efficiency, R^2, melting temperature Tm, the Y-Intercept and Cq values of negative controls (Cq neg. “-”equivalent to undetectable) are given for each run. *According to the melting curve analysis the Cq result for the negative control did not result from a contamination by the specific amplicon. (PDF) [file pone.0176287.s008.pdf]

		Slope	Efficiency	R^2	Tm	Y-Intercerpt	Cq neg.
**UCA1**	Run 1	-3.67	1.74	0.99	78.58	28.65	-
	Run 2	-3.41	1.92	0.99	78.42	27.43	-
	Run 3	-3.59	1.79	0.98	78.59	28.12	-
**MALAT1**	Run1	-3.81	1.83	0.99	81.35	27.39	-
	Run2	-3.31	2.00	1.00	81.35	26.47	-
	Run3	-3.33	2.00	1.00	81.36	26.53	-
**GAS5**	Run1	-3.29	2.01	1.00	79.01	29.31	-
	Run2	-3.43	1.99	1.00	79.17	29.51	-
	Run3	-3.22	2.04	1.00	79.14	28.84	-
**H19**	Run1	-3.32	2.00	1.00	86.69	27.54	-
	Run2	-3.39	1.97	1.00	86.79	27.60	-
	Run3	-3.27	2.02	1.00	86.65	29.02	-
**TUG1**	Run1	-3.91	1.80	0.98	80.90	32.43	-
	Run2	-3.24	2.03	1.00	80.94	30.75	37.81
	Run3	-3.27	2.02	2.00	80.89	30.76	-
**ncRAN**	Run1	-3.59	1.90	0.97	84.42	28.48	-
	Run2	-3.29	2.01	1.00	84.42	27.42	35.81
	Run3	-3.23	2.04	1.00	84.41	27.38	34.49
**linc-UBC1**	Run1	-3.28	2.02	0.99	78.20	35.56	-
	Run2	-3.24	2.03	1.00	78.20	35.72	-
	Run3	-3.49	1.95	0.95	78.14	35.55	36.79*
**TBP**	Run1	-3.21	2.05	0.99	83.39	33.16	-
	Run2	-3.61	1.89	0.96	83.39	34.19	-
	Run3	-3.13	2.09	0.96	83.34	33.51	-
**SDHA**	Run1	-3.37	1.98	1.00	80.73	31.98	-
	Run2	-3.27	2.02	0.99	80.75	31.79	-
	Run3	-3.66	1.88	0.99	80.87	32.54	-
