# Supplementary material for: Diagnostic and prognostic value of long noncoding RNAs as biomarkers in urothelial carcinoma
Source: PLoS One. 2017 Apr 21;12(4):e0176287. doi: 10.1371/journal.pone.0176287 (PMC5400278; doi:10.1371/journal.pone.0176287)
Supplement: S3 Table — For the second reverse transcription of tissue sample RNA RT-qPCR runs were conducted with normal and tumor samples distributed equally. Information on slope of the standard curve, resulting efficiency, R^2, melting temperature Tm, the Y-Intercept and Cq values of negative controls (Cq neg. “-”equivalent to undetectable) are given for each run. *According to the melting curve analysis the Cq result for the negative control did not result from a contamination by the specific amplicon. (PDF) [file pone.0176287.s009.pdf]

		Slope	Efficiency	R^2	Tm	Y-Intercerpt	Cq neg
**TBP**	Run 1	-3.15	2.08	1.00	83.60	32.50	35.91*
	Run 2	-3.37	1.98	1.00	83.61	32.74	34.39*
	Run 3	-3.50	1.99	0.99	83.66	32.76	35.51*
	Run 4	-3.21	2.05	0.98	83.60	32.61	35.68*
	Run 5	-3.40	1.97	0.99	83.63	32.88	35.79*
**SDHA**	Run 1	-3.38	1.98	1.00	80.98	30.66	37.65*
	Run 2	-3.35	1.99	1.00	81.01	30.51	-
	Run 3	-3.39	1.97	0.99	80.96	30.69	-
	Run 4	-3.21	2.05	1.00	81.02	30.52	37.3*
	Run 5	-3.40	1.97	1.00	80.96	31.04	-
**Ki67**	Run 1	-3.31	2.01	1.00	81.25	29.49	-
	Run 2	-3.41	1.97	0.99	81.27	29.45	-
	Run 3	-3.44	1.95	1.00	81.29	29.60	37.15
	Run 4	-3.39	1.97	1.00	81.23	29.58	-
