# Supplementary material for: Diagnostic and prognostic value of long noncoding RNAs as biomarkers in urothelial carcinoma
Source: PLoS One. 2017 Apr 21;12(4):e0176287. doi: 10.1371/journal.pone.0176287 (PMC5400278; doi:10.1371/journal.pone.0176287)
Supplement: S4 Table — Hazard Ratios (HR) with a 95% Confidence Interval (CI) and p-values (P) were calculated by Cox regression analyses on overall and disease-specific survival for lncRNA expression levels for all patients with T2-T4 tumors in set 1. Patients were divided into a low- and a high-expression group for each lncRNA by median expression. The cut-off is based on the whole Ta-T4 cohort. Bold printed p-values were significant (≤0.05). (PDF) [file pone.0176287.s010.pdf]

| Variables | Overall survival | | | Disease-Specific survival | | |
|---|---|---|---|---|---|---|
| | HR | 95% CI | P | HR | 95% CI | P |
| **UCA1 exp. 50%** | | | | | | |
| **low** | ref. | | | ref. | | |
| **high** | 0.565 | 0.347-0.922 | **0.022** | 0.592 | 0.349-1.005 | 0.052 |
| **UBC1 exp. 50%** | | | | | | |
| **low** | ref. | | | ref. | | |
| **high** | 0.886 | 0.544-1.422 | 0.626 | 0.978 | 0.578-1.657 | 0.935 |
| **TUG1 exp 50%** | | | | | | |
| **low** | ref. | | | ref. | | |
| **high** | 0.621 | 0.380-1.016 | 0.058 | 0.642 | 0.377-1.093 | 0.103 |
| **ncRAN exp. 50%** | | | | | | |
| **low** | ref. | | | ref. | | |
| **high** | 0.979 | 0.602-1.592 | 0.933 | 0.758 | 0.442-1.298 | 0.313 |
| **MALAT1 exp. 50%** | | | | | | |
| **low** | ref. | | | ref. | | |
| **high** | 0.621 | 0.377-1.021 | 0.060 | 0.675 | 0.395-1.154 | 0.151 |
| **H19 exp. 50%** | | | | | | |
| **low** | ref. | | | ref. | | |
| **high** | 0.936 | 0.577-1.519 | 0.789 | 0.981 | 0.580-1.659 | 0.944 |
| **GAS5 exp. 50%** | | | | | | |
| **low** | ref. | | | ref. | | |
| **high** | 0.754 | 0.462-1.232 | 0.260 | 0.767 | 0.450-1.305 | 0.328 |
