# Supplementary material for: Diagnostic and prognostic value of long noncoding RNAs as biomarkers in urothelial carcinoma
Source: PLoS One. 2017 Apr 21;12(4):e0176287. doi: 10.1371/journal.pone.0176287 (PMC5400278; doi:10.1371/journal.pone.0176287)
Supplement: S5 Table — UCA1, TUG1, ncRAN and MALAT1 expression had a statistically significant impact on patient overall or disease-specific survival and were further analysed in the multivariate analyses including tumor stage, grade and lymph node metastasis as parameters. Hazard Ratios (HR) with a 95% Confidence Interval (CI) and p-values (p) are given for overall and disease-specific survival. Bold printed p-values were significant (≤0.05). (PDF) [file pone.0176287.s011.pdf]

	Overall survival			Disease-specific survival		
**Variables**	**HR**	**95% CI**	**p**	**HR**	**95% CI**	**p**
**All cases (Ta-T4)**						
**Stage (T3-T4)**	0.790	0.437-1.429	0.435	0.815	0.423-1.570	0.542
**Grade (G3)**	1.788	0.889-3.598	0.103	2.132	0.939-4.840	0.070
**Lymph node metastasis (N+)**	2.490	1.434-4.324	**0.001**	3.022	1.678-5.440	**<0.001**
**UCA1 (> 50%)**	0.606	0.368-0.998	**0.049**	0.648	0.377-1.112	0.116

	Overall survival			Disease-specific survival		
**Variables**	**HR**	**95% CI**	**p**	**HR**	**95% CI**	**p**
**All cases (Ta-T4)**						
**Stage (T3-T4)**	0.754	0.416-1.367	0.352	0.794	0.412-1.531	0.491
**Grade (G3)**	1.772	0.870-3.608	0.115	2.090	0.910-4.803	0.082
**Lymph node metastasis (N+)**	2.679	1.550-4.630	**<0.001**	3.210	1.793-5.746	**<0.001**
**TUG1 (> 50%)**	0.600	0.362-0.993	**0.047**	0.629	0.364-1.085	0.095

	Overall survival			Disease-specific survival		
**Variables**	**HR**	**95% CI**	**p**	**HR**	**95% CI**	**p**
**All cases (Ta-T4)**						
**Stage (T3-T4)**	0.750	0.414-1.360	0.344	0.805	0.418-1.552	0.518
**Grade (G3)**	2.037	0.997-4.160	0.051	2.194	0.952-5.057	0.065
**Lymph node metastasis (N+)**	2.498	1.451-4.298	**0.001**	3.082	1.727-5.052	**<0.001**
**ncRAN (> 50%)**	1.063	0.644-1.755	0.811	0.814	0.466-1.419	0.467

	Overall survival			Disease-specific survival		
**Variables**	**HR**	**95% CI**	**p**	**HR**	**95% CI**	**p**
**All cases (Ta-T4)**						
**Stage (T3-T4)**	0.715	0.395-1.297	0.270	0.756	0.392-1.460	0.405
**Grade (G3)**	1.996	0.997-3.995	0.051	2.344	1.039-5.290	**0.040**
**Lymph node metastasis (N+)**	2.423	1.408-4.169	**0.001**	2.949	1.653-5.262	**<0.001**
**MALAT1 (> 50%)**	0.623	0.377-1.030	0.065	0.700	0.407-1.203	0.196
