# Supplementary material for: Diagnostic and prognostic value of long noncoding RNAs as biomarkers in urothelial carcinoma
Source: PLoS One. 2017 Apr 21;12(4):e0176287. doi: 10.1371/journal.pone.0176287 (PMC5400278; doi:10.1371/journal.pone.0176287)
Supplement: S6 Table — Hazard Ratios (HR) with a 95% Confidence Interval (CI) and p-values (P) were calculated by Cox regression analyses on overall and disease-specific survival for lncRNA expression levels in set 1. Patients were divided into a low- and a high-expression group for each lncRNA by median expression. Bold printed p-values were significant (≤0.05). (PDF) [file pone.0176287.s012.pdf]

Variables	Overall survival		
	**HR**	**95% CI**	**P**
**Age**			
**≤ 65**	ref.		
**> 65**	0.67	0.377-0.861	**0.008**
**Sex**			
**Female**	ref.		
**Male**	1.15	0.743-1.781	0.529
**Lymph node status**			
**pNx / N0**	ref.		
**pN+**	2.12	1.433-3.137	**0.000**
**ncRAN exp. 50%**			
**low**	ref.		
**high**	1.53	1.036-2.249	**0.032**
**MALAT1 exp. 50%**			
**low**	ref.		
**high**	0.67	0.558-0.99	**0.044**
