# Supplementary material for: Diagnostic and prognostic value of long noncoding RNAs as biomarkers in urothelial carcinoma
Source: PLoS One. 2017 Apr 21;12(4):e0176287. doi: 10.1371/journal.pone.0176287 (PMC5400278; doi:10.1371/journal.pone.0176287)
Supplement: S1 File — (PDF) [file pone.0176287.s013.pdf]

Supplementary methods

Urothelial carcinoma tissue samples and benign controls were obtained from patients who underwent surgical treatment for UC, mainly subsequent to cystectomy. Selection and histological evaluation of the samples was performed by a qualified pathologist at the Department of Pathology. Samples were directly snap frozen in liquid nitrogen and kept at -80°C for longterm storage.

Tissue sections were stained with hematoxylin and eosin, only tumor tissues containing 70% or more tumour cells were selected for RNA extraction (S1 Fig). For further characterization as tumour tissue RNA expression of *MKi67* was determined (S1 Fig). TCGA samples contained at least 60% tumour cells as they found that "60% was sufficient to generate high quality data in which the tumor's signal could be distinguished from other cells' signals." (https://cancergenome.nih.gov/cancersselected/biospeccriteria). Patients of both cohorts had not received neoadjuvant treatment.

Tissue pieces were processed into powder by means of mortar and pistil under liquid nitrogen. RNA was extracted as described in the main text. Purity, quantity and quality were determined by photometric devices (Photometer, Nanodrop), by agarose gels and Agilent Bioanalyzer. RNA was stored at -80°C.

RNA was reverse transcribed as described in the main text. cDNA synthesis for set 1 was performed by means of the High Capacity cDNA Reverse Transcription Kit with 3 µg RNA in a 30 µl reaction volume according to the manufacturer. This kit includes random primers. For UC cell lines 1 µg RNA was reverse transcribed in a 20 µl reaction volume by using the Quantitect Reverse Transcription Kit including a mixture of random and oligo dT primers.

cDNAs were 1:10 diluted with PCR grade water. 2 µl diluted cDNA was added to a 20 µl reaction volume for qPCR. The efficiency of the reverse transcription was checked by initial measurements of the reference genes *TBP* and *SDHA*. Suitability of different reference genes across different tissues has been investigated by other groups in the past [22]. According to those results and our experiences over the last decade we used *TBP* and *SDHA* as reference genes, which appeared to be stably and equally expressed across UC tissue samples in our measurements. Diluted cDNA samples of satisfying quality were usually characterized by Cq values for TBP no higher than 28 cycles.

qPCR primers were self-designed or taken from the literature. All primer sequences were checked *in silico* for specificity by a BLAST analysis. For each assay, we performed a number of test runs across different cell line and tissue samples to determine specificity and the range of expression across different samples. RT minus controls without the addition of reverse transcriptase were included to check for contamination by genomic DNA (S6 Fig). Preferably cell line samples with robust expression were chosen as a reference sample for standard curves to determine PCR efficiency, linear dynamic range, detection limit etc. A melting curve analysis was performed to assure that only one PCR product was amplified and to check for primer dimers. Additionally, selected samples were loaded onto an agarose gel to further visualize amplification of a single product, and selected samples were purified and analyzed by Sanger sequencing. Sequencing results were verified by BLAST analysis. In every run measuring expression in tissue samples, an internal standard curve was included for relative quantification and to adjust for inter-assay variation. Relative expression of the target genes was determined by calculating the ratio between the relative quantification values for the target gene and the calculated normalization factor for the two reference

genes (see main text). We set our own internal cut-off for variation in terms of concentration ≤ 10 %. Assays with higher variation were repeated in another run.
